# Antioxidant Gene Overexpression Enhances Salt Tolerance in Transgenic Peppermint (*Mentha × piperita* L.)

**DOI:** 10.3390/ijms272110035

**Published:** 2026-11-09

**Authors:** Natalia O. Yuorieva, Galina N. Raldugina, Roman A. Sidorov, Pavel A. Syromyatnikov, Helen I. Rostovtseva, Liliya R. Bogoutdinova, Vyacheslav V. Latushkin, Lev N. Radchenko, Joseph M. Mikhel, Ekaterina V. Tkacheva, Olga V. Shelepova, Alexander A. Gulevich, Ludmila V. Kurenina, Ekaterina N. Baranova

**Affiliations:** 1 K.A. Timiryazev Institute of Plant Physiology, Russian Academy of Sciences, Botanicheskaya St., 35, Moscow 127276, Russia; yuorieva@mail.ru (N.O.Y.); roman.sidorov@mail.ru (R.A.S.); shout.cheers@gmail.com (P.A.S.); ni-fir-titi@mail.ru (H.I.R.); 2 All-Russia Research Institute of Agricultural Biotechnology, Timiryazevskaya 42, Moscow 127550, Russia; bogoutdinova_lr@rambler.ru (L.R.B.); 88888888vosem@gmail.com (L.N.R.); joseph.mikhel@gmail.com (J.M.M.); a_gulevich@mail.ru (A.A.G.); ludmila.kur2208@gmail.com (L.V.K.); 3 N.V. Tsitsin Main Botanical Garden of Russian Academy of Sciences, Botanicheskaya 4, Moscow 127276, Russia; gbsad_lib@mail.ru (E.V.T.); shov_gbsad@mail.ru (O.V.S.); 4 Institute of Development Strategy, Moscow 101000, Russia; slavalat@yandex.ru

**Keywords:** peppermint, in vitro, salinity, ROS, transgenic plants, Fe-dependent superoxide dismutase, choline oxidase, salt tolerance, glycophyte

## Abstract

High-menthol mint varieties are traditionally grown in regions characterized by a continental climate, characterized by pronounced seasonality with cold winters and hot, usually dry summers. Climate change means that this plant is increasingly grown in extreme conditions, where primary and secondary soil salinization is the norm. However, by its origin, mint is a typical glycophyte, that is, a plant that is very sensitive to the effects of salt, suspending the gain of biomass under conditions of increasing salinity and limited available water. We demonstrate an attempt to desensitize and increase tolerance of peppermint to high-salinity conditions corresponding to 100 mM and 150 mM NaCl. To maintain productivity under these uncharacteristic conditions, it was necessary to modify plant metabolism in such a way as to effectively utilize the non-specific potential inherent in plant genetics for resistance to stress factors characteristic of other abiotic stresses, such as cold or drought, which cause oxidative and osmotic damage to metabolism and growth. By obtaining peppermint plants transgenic for *Fe-SOD* and *codA* genes, we intended to induce mild internal stress, inducing protective mechanisms, without damaging the systems that ensure resistance due to specific anti-stress systems. An in vitro experiment confirmed that with this strategy, despite a significant decrease in growth and biomass, a decrease in the pool of photosynthetic pigments, and changes in the morphology of epidermal cells, the parameters recording plant viability were significantly higher in transgenic lines than in the original peppermint genotype. We also confirmed the absence of significant growth inhibition in transgenic lines under optimal conditions. This method of inducing non-specific resistance will be quite effective, but only if the salt concentration is subsequently reduced, since it is difficult to achieve a fundamental shift, manifested in the complete removal of the effects of stress in such a highly sensitive glycophyte as mint.

## 1. Introduction

Peppermint (*Mentha* × *piperita*) is a natural hybrid valued for its highly effective properties due to the unique composition of its essential oil. This essential oil is used in medicine, food production, cosmetology, and in the production of goods for which the presence of odor is important [1]. Different varieties of this hybrid form are of European origin and are characterized by high menthol content, but are now cultivated worldwide [2]. The global growth in demand for essential oil has generated interest in highly efficient technologies for breeding and producing mint planting material, driven by growing trends in demand for plant materials [3]. As with other vegetatively propagated plants such as potatoes, bamboo, grapes and many others, the problem of the impossibility of using seed reproduction requires careful adherence to technology to preserve properties and prevent loss of yield [4,5]. Meanwhile, the production of this popular culture is facing constraints caused by environmental changes [6,7]. Climate change is introducing new challenges to the productivity and sustainability of this popular crop. Producers are faced with the challenge of making a sound economic choice for growing this crop, which inherently lacked tolerance to adverse environmental factors as increased salinity and/or drought [8,9].

Tolerance to abiotic stresses is often provided by non-specific systems associated with resistance to other stress factors [10]. Abiotic stresses induce internal stress responses that manifest within cells and tissues as oxidative, toxic, and osmotic stress.

It has been previously shown that although mint is sensitive to moderate salinity, this did not affect the composition of the essential oil [11,12]. This gives hope that the synthesis of menthol under stressful conditions will not change towards the synthesis of compounds toxic to humans, such as pulegone [13]. Another problem is that reliable devolution of the properties of a particular genotype can only be ensured through vegetative propagation, since hybrid forms do not produce high-quality, aligned seeds and do not guarantee offspring with the desired characteristics. In this regard, routine technologies of green cuttings, segmentation of rhizomes and in vitro micropropagation have recently become the focus of research in the compilation of new technological maps for farms specializing in the cultivation of this crop [14,15]. Increasing tolerance and assessing resistance to drought and salinity, as the main critical production risks, have recently become focal research areas for the technology of the reproduction cycle, which takes about four years [8,9]. Modulation of gene expression using heterologous expression is currently an effective method for studying the relationship between stress resistance and stress responses at the cellular level. Photosynthetic tissues can act as a highly sensitive model with characteristic features of response to salinity. To mimic oxidative and osmotic priming in plastids, we used transgenesis with the *FeSOD* [16] and *codA* [17] genes, which cause an increase in the production of peroxide and glycine betaine, which is associated with oxidative and osmotic modifications in plastid compartments [18]. Choline oxidase, which is responsible for the synthesis of glycine betaine, also produces H_2_O_2_ as a by-product [19]. Due to the increase in the amount of peroxide, other antioxidant enzymes are activated [20]. Moreover, hydrogen peroxide is a common intracellular signaling molecule that triggers various protective processes [21,22].

In this study, we attempted to model the effects of oxidative and osmotic signaling through the induction of internal stress to identify changes in the tolerance of mint plants to moderate salinity. The goal of this work was to verify that these modifications alter plant metabolism under normal conditions and influence plant tolerance to salinity.

## 2. Results

### 2.1. Production of Transgenic Plants

Aseptically grown and micropropagated Mitcham peppermint plants (Figure S1a,b) were subjected to Agrobacterium-mediated transformation (Figure S1c–f). For this purpose, stem fragments (Figure S1c) and leaf explants (Figure S1d) were used. Since stem explants showed weak regeneration, only leaf explants were subsequently used (Figure S1e,f). After selection on kanamycin, transformed shoots were transferred to MS agar medium for rooting (Figure S1d). Ten independent lines transformed by the Fe-SOD gene and ten independent lines transformed with the *codA* gene were selected. Plants without visible morphogenesis defects were then subjected to molecular analysis to confirm the presence of the target genes and their expression (Figure S2). The plantlets were then propagated to study morphological and physiological parameters (Figure S1h).

### 2.2. The Effect of NaCl on the Development of Transgenic Lines in In Vitro Culture

As noted in the Introduction, mint is inherently a typical glycophyte—a plant highly sensitive to salt exposure; increased salinity and a lack of available moisture slow down or halt its biomass accumulation [11,23], thereby reducing the yield of essential oils, the primary objective of cultivating this plant.

In an effort to reduce peppermint’s sensitivity to salinity, we introduced the *FeSOD* and *codA* genes—which have previously been shown to enhance stress tolerance in other plant species [18,20,24,25]—into mint plants and subsequently investigated the effect of NaCl on the selected lines under in vitro conditions.

### 2.3. Effect of Elevated NaCl Concentrations on Morphometric Parameters of Transgenic Lines of Mint

Two concentrations were used that reversibly inhibited the growth and development of plants: 100 mM and 150 mM (Figure S5).

The latter completely inhibited the growth of roots and green parts, leaving the plants in a kind of dormant state, without causing death for a month and allowing growth to resume after washing and placing in optimal conditions. When cultivated for longer periods, more than 5–6 weeks, such explants died; thus, this concentration should be considered sublethal. Significant inhibition of root and stem growth was observed in both the transgenic lines and the original (control) genotype. Root growth at 100 mM NaCl was reduced more than twofold in the original genotype and fourfold in *codA*-transgenic plants. At 150 mM NaCl, it was retained only in FeSOD-transgenic lines at 100 mM; stem growth was significantly higher in plants overexpressing *FeSOD* and one of the *codA*-transgenic lines (CodA 8). Stem growth was completely inhibited at 150 mM and remained at the level of the original explants, with plantlets being slightly taller in the original genotype and the FeSOD11 line.

**Figure 1:**
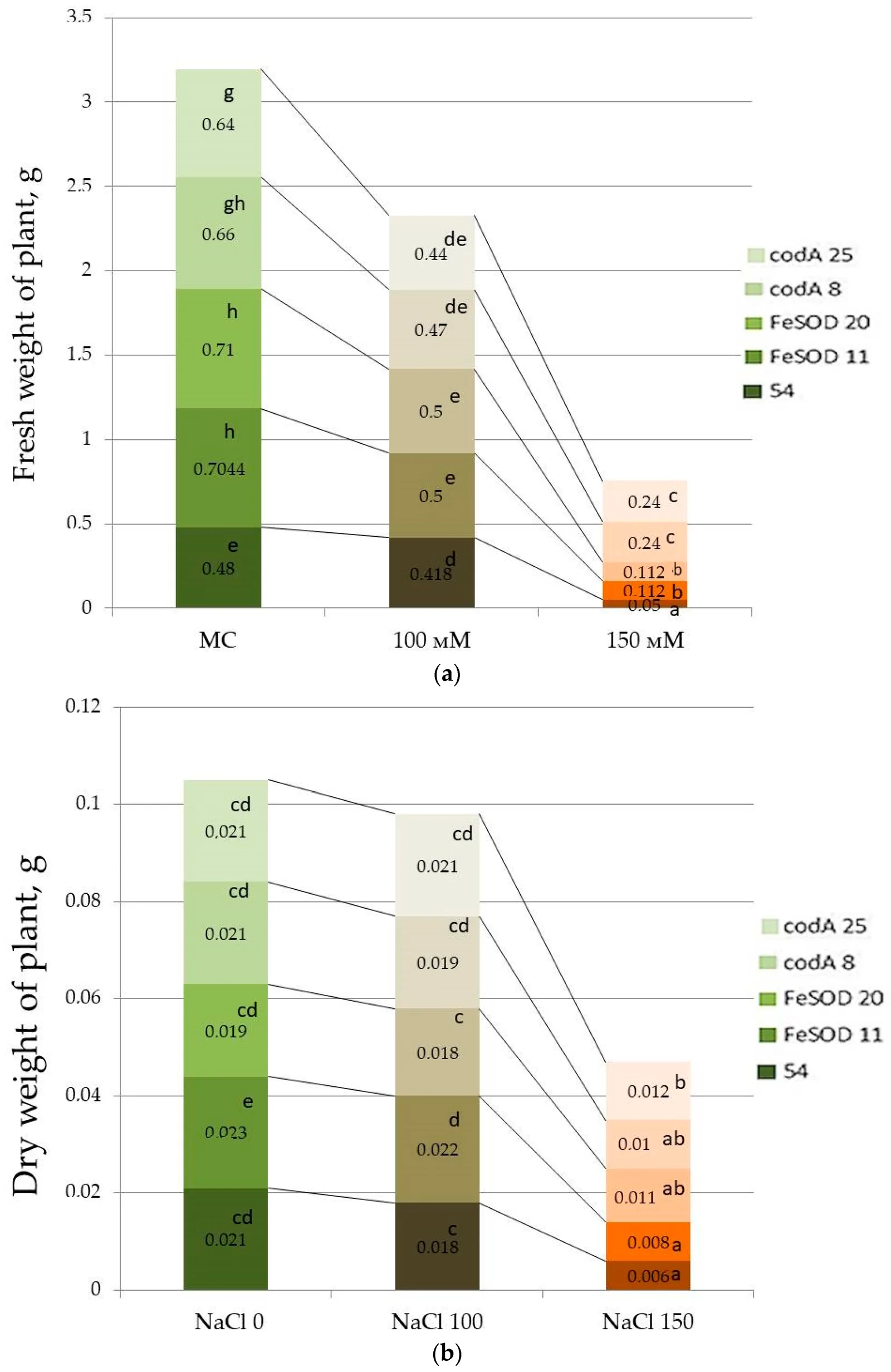
Change in the fresh (**a**) and dry (**b**) weight of in vitro mint plants under salt concentrations of 100 and 150 mM NaCl. In the legend, the corresponding accessions are indicated by a staining intensity similar to that observed under conditions without NaCl treatment. Different letters indicate significant differences between treatment, analyzed by a Duncan’s tests with the corresponding errors (*p* = 0.05).

Transgenic plants expressing *FeSOD* and *codA* had 1.5-fold greater biomass in vitro after four weeks. Salinity significantly reduced plant growth in both the wild-type and transgenic plants, with the *FeSOD*-transgenic plants exhibiting a somewhat smaller reduction (Figure 1). This trend was observed at both 100 mM and 150 mM NaCl. At the same time, plantlets showed virtually no growth at 150 mM, and with longer cultivation, the wild-type genotype died by day 50, while the transgenic plants remained viable.

**Figure 2:**
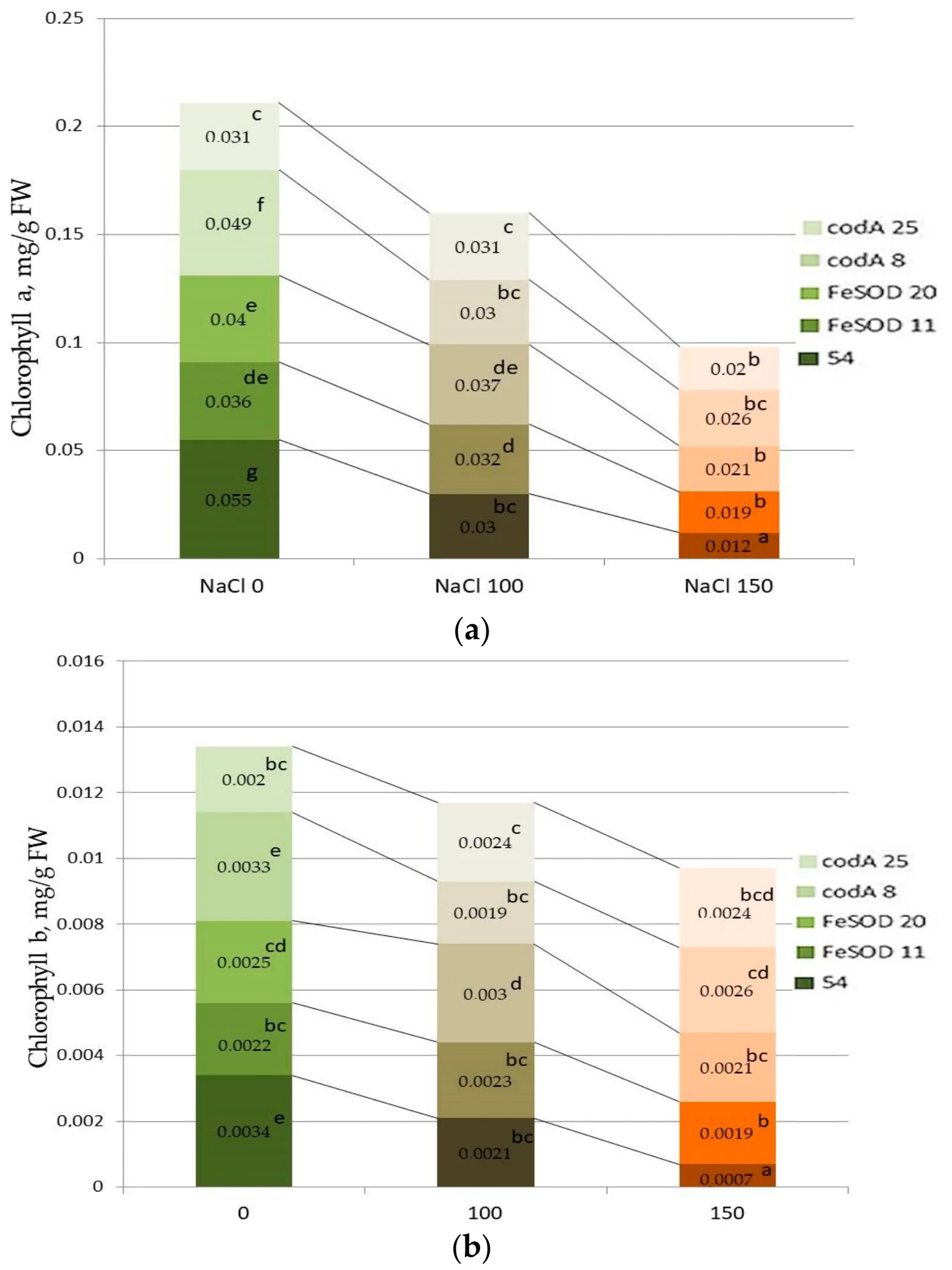
Changes in (**a**) chlorophyll a and (**b**) chlorophyll b content in response to salt concentrations of 100 and 150 mM NaCl in wild-type mint plants and *FeSOD* and *codA*-transgenic lines. In the legend, the corresponding accessions are indicated by a staining intensity similar to that observed under conditions without NaCl treatment. Different letters indicate significant differences between treatment, analyzed by a Duncan’s tests with the corresponding errors (*p* = 0.05).

A decrease in dry biomass was also observed, but it was less significant than the drop in fresh weight. Particularly at 100 mM NaCl, only the *codA*-transgenic line significantly maintained higher values than the wild-type mint genotype. As the concentration increased to 150 mM, dry biomass accumulation declined dramatically for all genotypes, falling by half even in the slightly more tolerant *FeSOD*-transgenic lines (Figure 2).

### 2.4. Effect of Elevated NaCl Concentrations on Physiological Parameters of Transgenic Lines of Mint

To adapt to salt stress, plants employ a variety of biochemical mechanisms. These include the selective accumulation or exclusion of ions; control over ion uptake by roots and their transport to leaves; ion compartmentalization at both cellular and whole-plant levels; synthesis of compatible osmolytes; alteration of photosynthesis; modification of membrane structure; and a number of others.

The content of the key photosynthesis enzyme, chlorophyll a, was reduced in all transgenic lines, but, unlike the wild-type genotype, a less significant decrease in this pigment was observed when cultivated in the presence of 100 mM NaCl. When cultivated in the presence of 150 mM NaCl, the decrease in chlorophyll a was almost fivefold in the wild-type genotype, while in the transgenic lines the decrease was no more than twofold (Figure 2a).

Chlorophyll b, which is more sensitive to stress, showed a significant decrease in wild-type plants both under 100 mM NaCl and in the presence of 150 mM NaCl. Meanwhile, chlorophyll b levels remained virtually stable in the transgenic lines, with the exception of the *FeSOD*-line, where they were comparable to the value under optimal conditions in the original genotype (Figure 2b).

**Figure 3:**
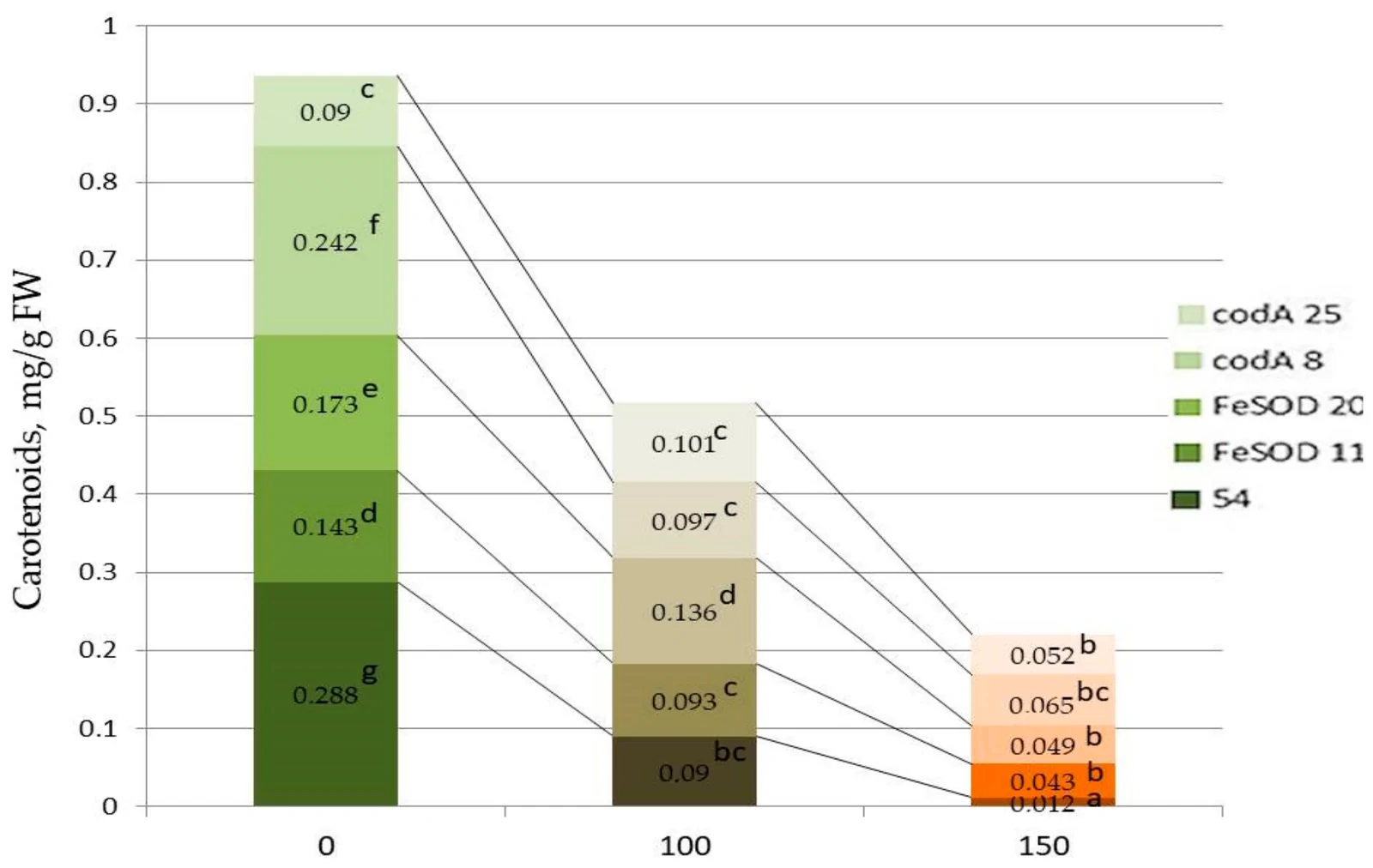
Changes in carotenoid content in response to salt concentrations of 100 and 150 mM NaCl in wild-type mint plants and *FeSOD*- and *codA*-transgenic lines. The corresponding accessions are indicated by a staining intensity similar to that observed under conditions without NaCl treatment. Different letters indicate significant differences between treatment, analyzed by a Duncan’s tests with the corresponding errors (*p* = 0.05).

Carotenoid levels in mint plants grown in vitro were higher in the wild-type genotype. Cultivation at both 100 mM NaCl and 150 mM NaCl resulted in a significant reduction in carotenoid content in the transgenic lines, but the decline was particularly dramatic in the wild-type, given the high carotene concentrations in the absence of treatment. Carotenoid levels were comparable at 100 mM NaCl and lower than those in all transgenic lines at 150 mM NaCl (Figure 3).

**Figure 4:**
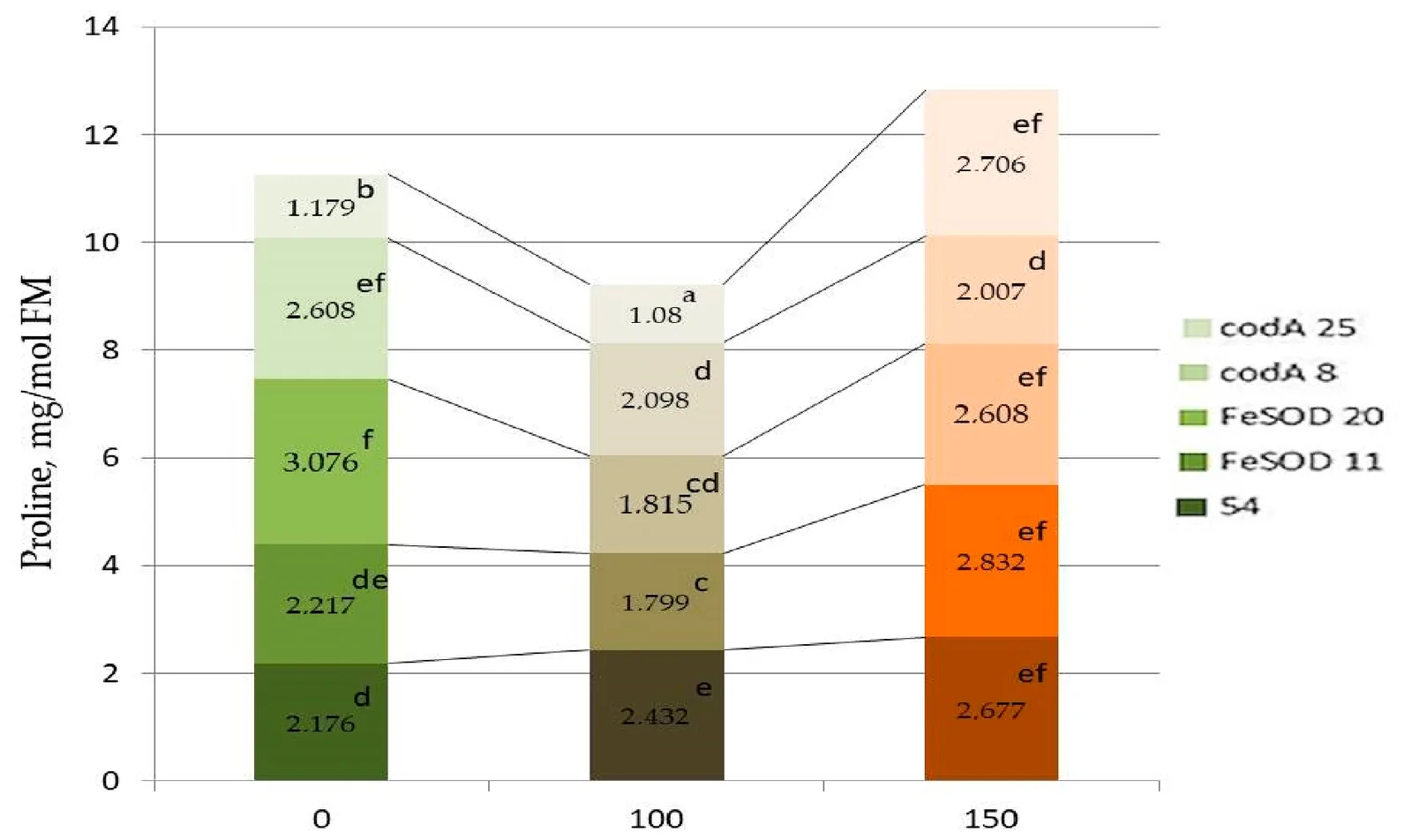
Changes in proline content in response to salt concentrations of 100 and 150 mM NaCl in wild-type mint plants and *FeSOD*- and *codA*-transgenic lines. The corresponding accessions are indicated by a staining intensity similar to that observed under conditions without NaCl treatment. Different letters indicate significant differences between treatment, analyzed by a Duncan’s tests with the corresponding errors (*p* = 0.05).

An increase in proline content was observed in the wild-type genotype at both salt concentrations. In the transgenic lines, however, we observed a decrease in concentration at 100 mM NaCl and a significant increase at 150 mM NaCl, comparable to the proline concentration in the wild-type, with the exception of the FeSOD11 line (Figure 4).

**Figure 5:**
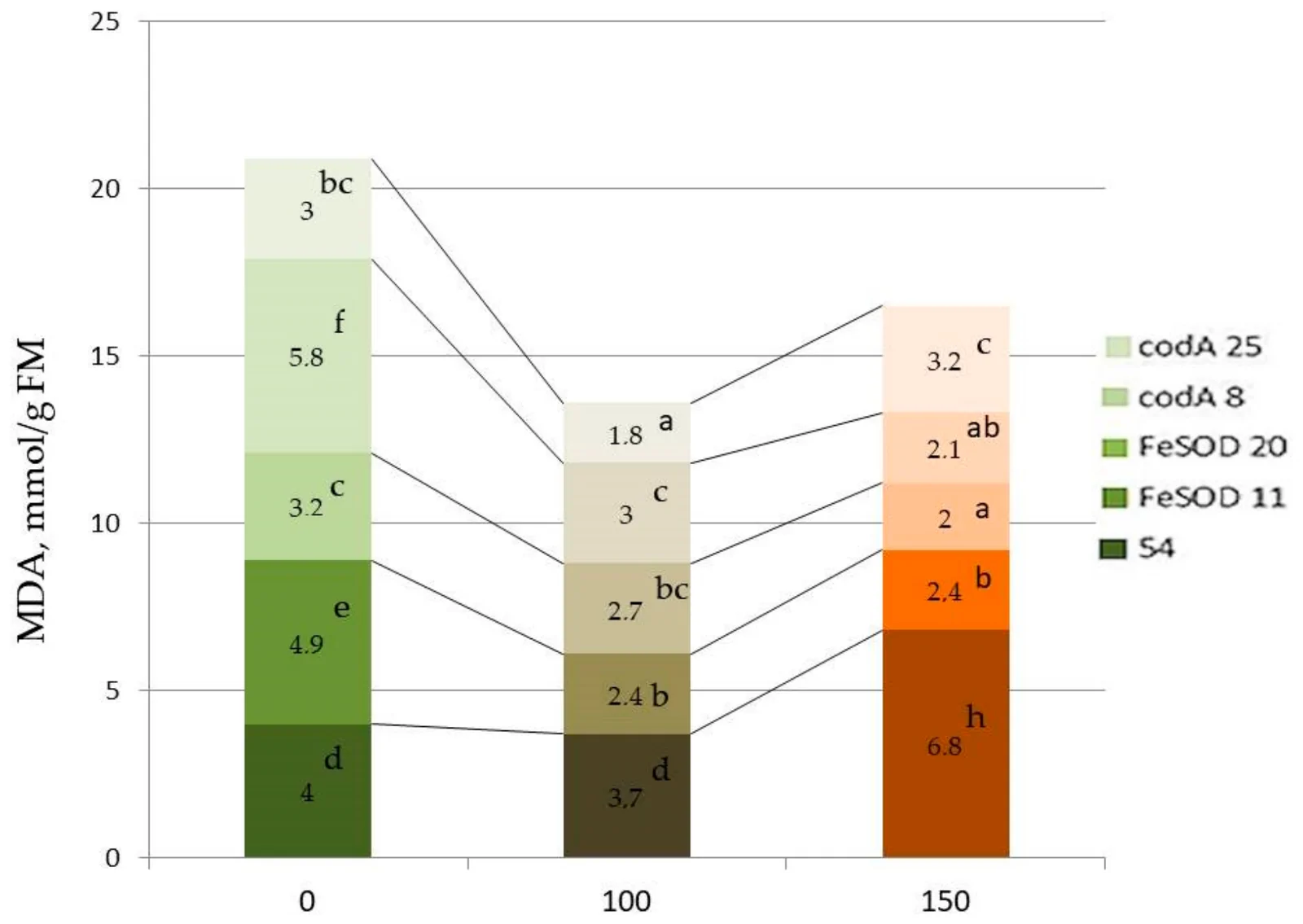
MDA content in response to salt concentrations of 100 and 150 mM NaCl in wild-type mint plants and *FeSOD*- and *codA*-transgenic lines. The corresponding accessions are indicated by a staining intensity similar to that observed under conditions without NaCl treatment. Different letters indicate significant differences between treatment, analyzed by a Duncan’s tests with the corresponding errors (*p* = 0.05).

Lipid peroxidation and membrane quality were assessed by malondialdehyde (MDA) accumulation. We observed that cultivation at 100 mM NaCl had no effect on MDA content in the wild-type genotype. However, under optimal conditions, transgenic plants exhibited variable MDA levels. Under 100 mM NaCl, MDA levels significantly decreased below those in the wild-type genotype and remained low even when exposed to 150 mM NaCl (Figure 5). In the wild-type genotype, MDA levels increased critically at high concentrations, demonstrating significant damage.

**Figure 6:**
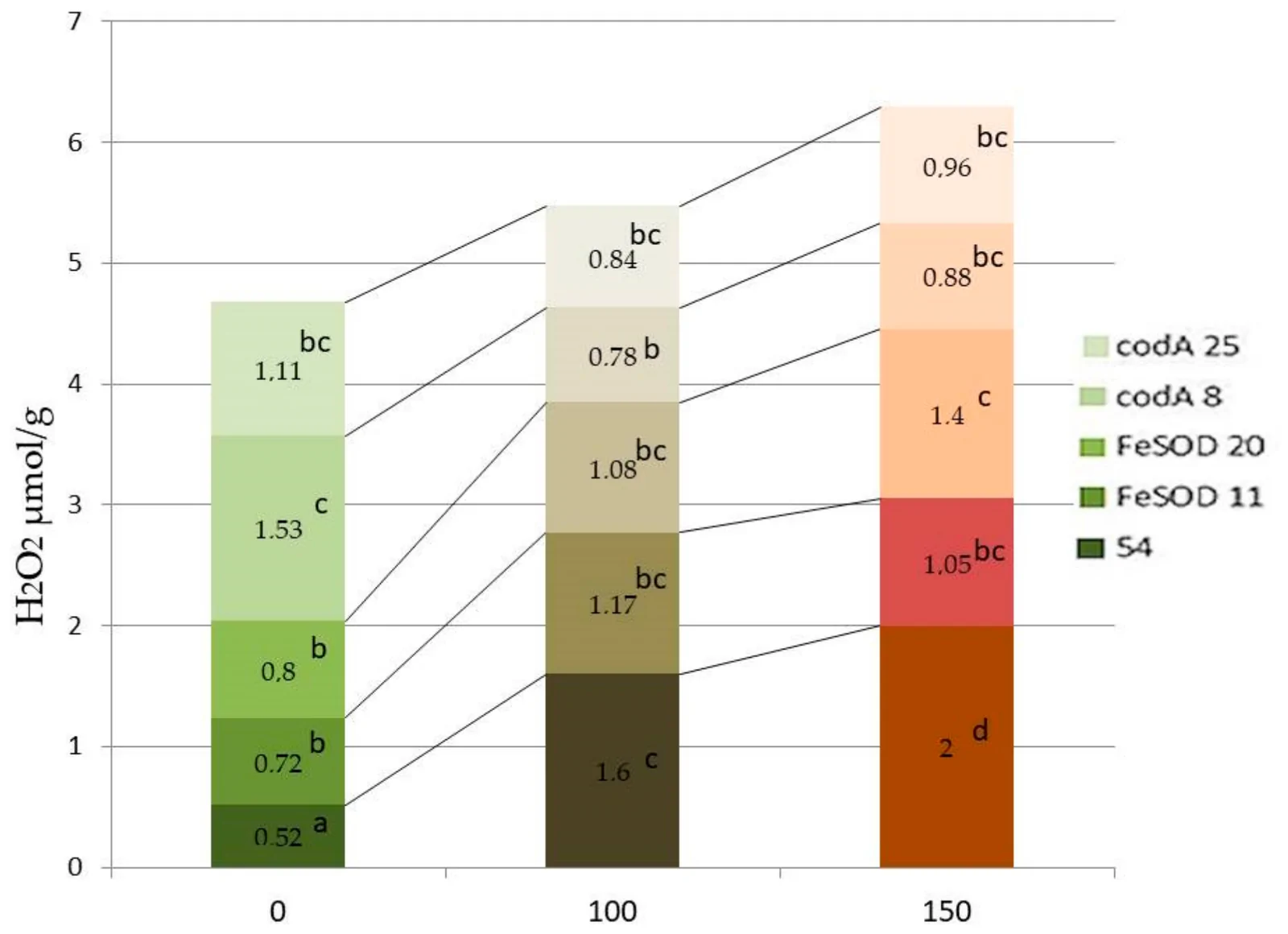
Hydrogen peroxide content in response to salt concentrations of 100 and 150 mM NaCl in wild-type mint plants and *FeSOD*- and *codA*-transgenic lines. The corresponding accessions are indicated by a staining intensity similar to that observed under conditions without NaCl treatment. Different letters indicate significant differences between treatment, analyzed by a Duncan’s tests with the corresponding errors (*p* = 0.05).

When cultivated in vitro on MS medium, the hydrogen peroxide content in transgenic plants was significantly higher than that of the wild-type genotype (Figure 6). In plants expressing *FeSOD*, this value was more than twice that of wild-type plants, while in *codA* transgenic plants, it was more than a third higher, but lower than that of *FeSOD*-expressing plants. At 100 mM NaCl, a threefold increase in this parameter was observed in the wild-type genotype, a decrease in *FeSOD*-expressing plants, and a tendency toward an increase in *codA*-transgenic plants.

Analysis of the primary response of barrier systems revealed an initially paradoxical pattern in the dynamics of the membrane injury index (Electrolyte leakage). Under control conditions, the studied samples exhibited high background values of electrolyte leakage, ranging from 44.9% (in 54MC) to a maximum of 61.9% (in FeSOD20). However, under salinity stress (100 mM NaCl), a sharp drop in total electrolyte leakage to minimal values was recorded, ranging from 2.4% in 54MC to 11.5–12.6% in CodA25 and FeSOD20.

This phenomenon indicates that the introduction of exogenous sodium into the external solution shifts the osmotic gradient and induces the activation of emergency plasmalemma stabilization systems in all studied genotypes. Nonetheless, the samples differed significantly in their ability to maintain barrier integrity. The genotype 54MC proved to be the most stable and resistant to membrane destruction, maintaining a minimal leakage level of 2.4%. In contrast, FeSOD20 (12.6%) and CodA25 (11.5%) exhibited the highest degree of damage to surface cellular barriers, rendering them potentially vulnerable to salt shock.

### 2.5. Effect of NaCl on Transgenic Lines of Mint in Soil Culture

Soil salinity is a major global challenge for mint cultivation, given that less valuable agricultural land is often used for growing aromatic plants. This phenomenon poses significant obstacles to mint cultivation and can affect plant growth, yield, and various metabolic processes, including lipid and secondary metabolism [26]. Therefore, we selected this stress factor to evaluate the tolerance of the generated transgenic lines. To assess this impact, we analyzed the lipid and essential oil composition in the leaves of selected transgenic mint lines grown in saline soil. Determining the fatty acid (FA) and essential oil (EO) composition of in vitro cultured plants proved difficult due to growth retardation at high NaCl concentrations and the resulting insufficient biomass accumulation. Rooted in vitro cuttings were transplanted into soil treated with a 100 mM NaCl solution, and samples were collected on the seventh day.

### 2.6. Dynamics of the Membrane Injury Index

Analysis of the primary response of barrier systems revealed an initially paradoxical pattern in the dynamics of the membrane injury index (Electrolyte leakage). Under control conditions, the studied samples exhibited high background values of electrolyte leakage, ranging from 44.9% (in 54MC) to a maximum of 61.9% (in FeSOD20). However, under salinity stress (100 mM NaCl), a sharp drop in total electrolyte leakage to minimal values was recorded, ranging from 2.4% in 54MC to 11.5–12.6% in CodA25 and FeSOD20.

**Figure 7:**
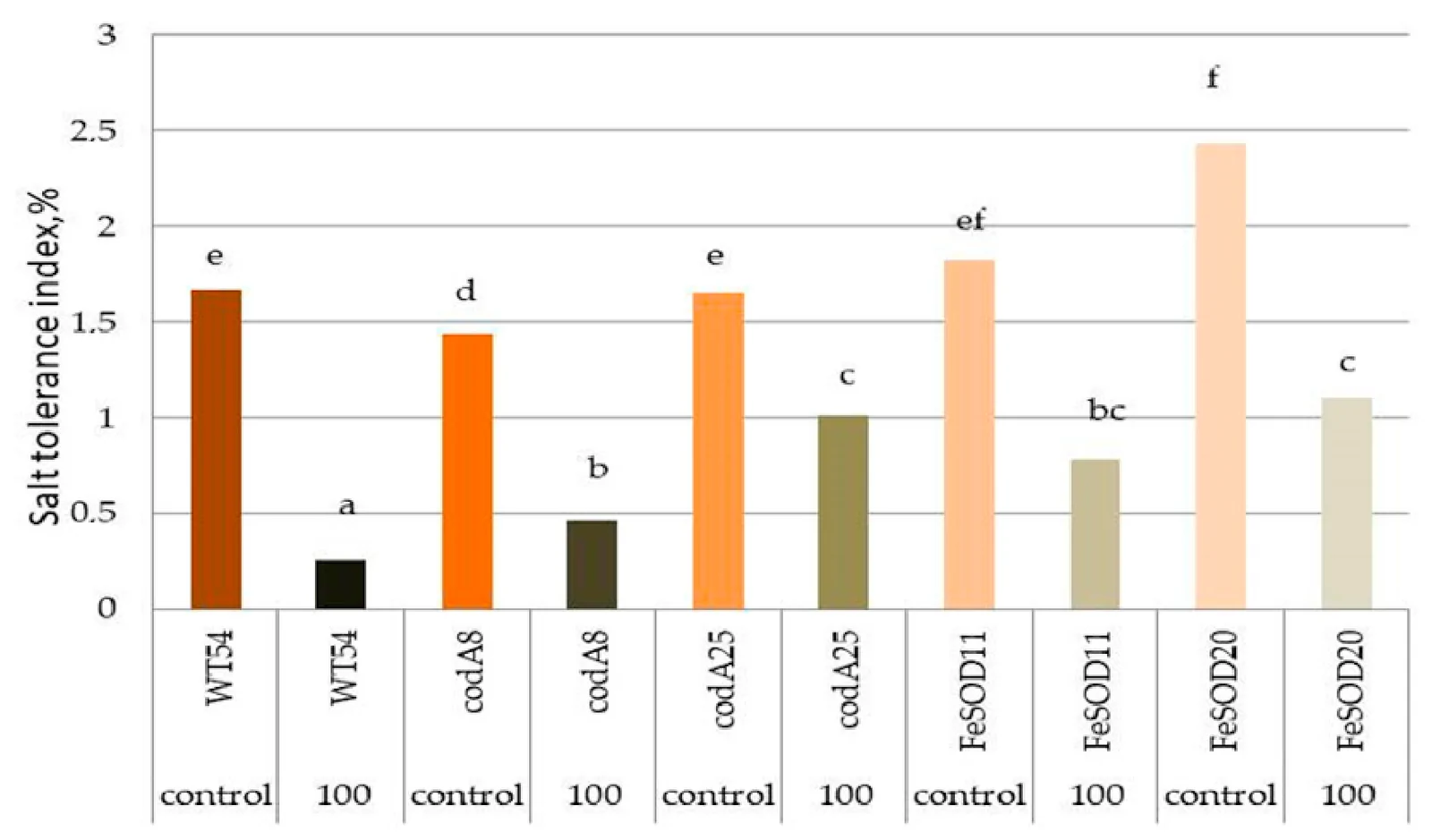
The index characterizing salt tolerance—the membrane injury index (electrolyte leakage)—of mint regenerant samples during comparative in vitro evaluation in wild-type mint plants and *FeSOD*- and *codA*-transgenic lines. Different letters indicate significant differences between treatment, analyzed by a Duncan’s tests with the corresponding errors (*p* = 0.05).

This phenomenon indicates that the introduction of exogenous sodium into the external solution shifts the osmotic gradient and induces the activation of emergency plasmalemma stabilization systems in all studied genotypes. Nonetheless, the samples differed significantly in their ability to maintain barrier integrity. The genotype 54MC proved to be the most stable and resistant to membrane destruction, maintaining a minimal leakage level of 2.4%. In contrast, FeSOD20 (12.6%) and CodA25 (11.5%) exhibited the highest degree of damage to surface cellular barriers, rendering them potentially vulnerable to salt shock (Figure 7).

The dynamics of the salt tolerance index revealed a direct correlation with the patterns of cation accumulation and redistribution. Under control conditions, the FeSOD20 genotype possessed the highest potential (2.43); however, under the influence of the saline agent, this index decreased by more than half to 1.07. The highest stability and capacity to maintain metabolism under stress was demonstrated by the sample CodA25, which maintained its salt index at 1.01. The genotype 54WT was found to be the most sensitive to salinity, as its index dropped to a minimum value of 0.27, indicating a sharp suppression of growth or biochemical parameters despite the preservation of physical membrane integrity noted above.

### 2.7. Cation Leakage Selectivity and K^+^/Na^+^ Ratio Salt Stress Impact

**Figure 8:**
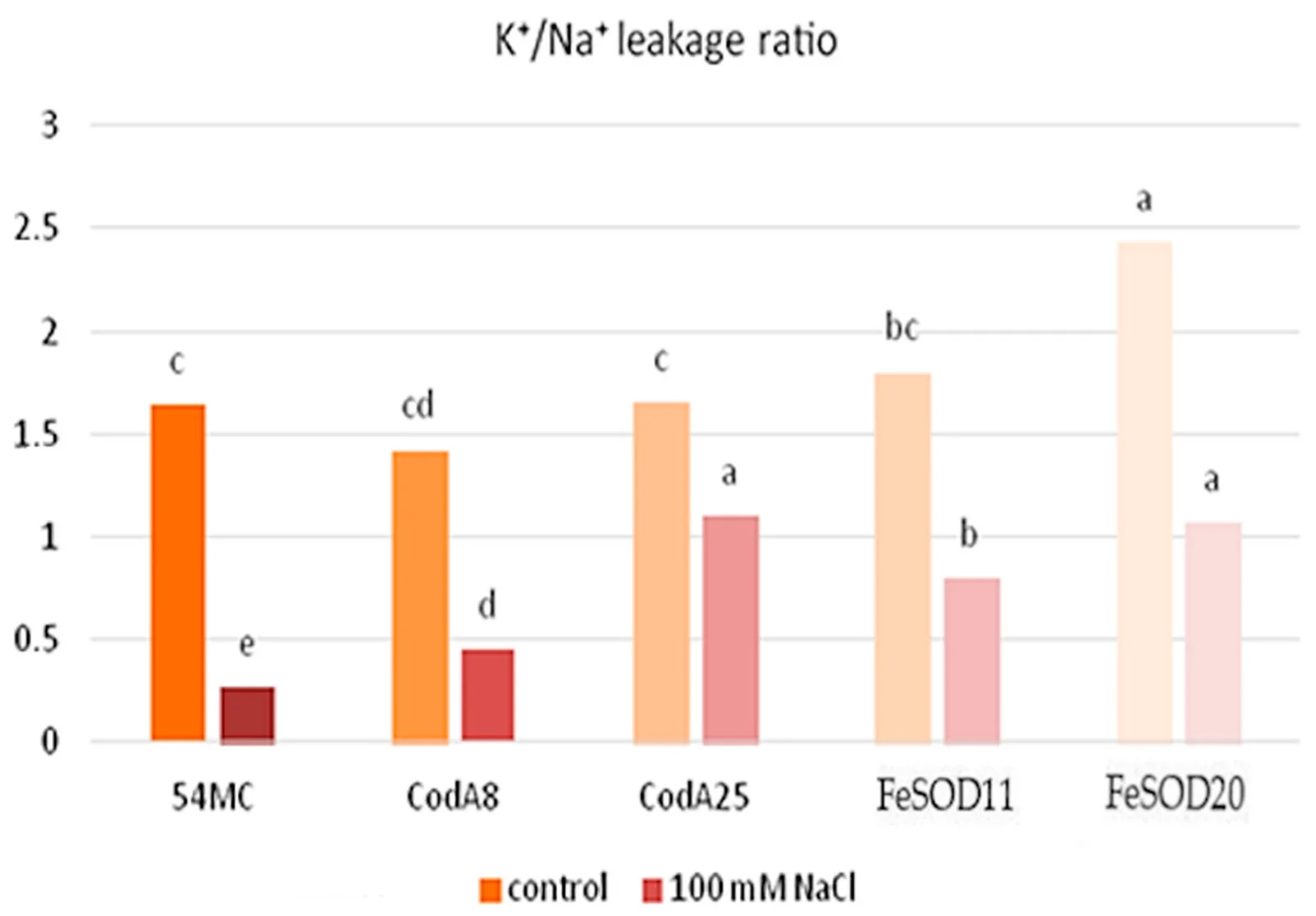
The index characterizing salt tolerance—the K^+^/Na^+^ leakage ratio of mint regenerant samples during comparative in vitro evaluation in wild-type mint plants and *FeSOD*- and *codA*-transgenic lines. The corresponding accessions are indicated by a staining intensity similar to that observed under conditions without NaCl treatment. Different letters indicate significant differences between treatment, analyzed by a Duncan’s tests with the corresponding errors (*p* = 0.05).

The ion leakage index reflects stress-induced damage to cellular transport systems. Under control conditions potassium was preferentially leaked from all samples (56.6–78.5%), while sodium efflux was tightly restricted (38.5–53.9%), maintaining the K^+^/Na^+^ leakage ratio above 1.0 (up to 2.43 in FeSOD20). Under 100 mM NaCl, a cascading efflux occurred, with Na^+^ leakage reaching critical levels (82.6–93.4%) and outpacing K^+^ leakage. Consequently, the K^+^/Na^+^ ratio dropped below 1.0 in most genotypes, particularly in 54MC (0.27) and CodA8 (0.45), indicating a severe cation imbalance and loss of channel selectivity. In contrast, CodA25 and FeSOD20 successfully maintained their K^+^/Na^+^ ratios above equilibrium at 1.10 and 1.07, respectively. This retention capacity suggests efficient operation of ion antiporters (such as NHX transporters) maintaining ionic homeostasis under severe saline pressure (Figure 8).

### 2.8. Determination of Fatty Acid Composition in Transgenic Mint Lines Under Elevated NaCl Concentration in Soil

Salt stress induced changes in the content of unsaturated and saturated fatty acids in the leaves of the transgenic forms studied, differing from the response of these parameters in the parental form.

Table 1 presents the fatty acid composition of total lipids in mint leaves across experimental variants and durations of salt stress exposure. The predominant fatty acids in all variants were palmitic, oleic, linoleic, and α-linolenic acids, collectively accounting for over 94% of the total fatty acids; minor fatty acids are listed in the table footnotes.

The wild-type line (54WT) exhibited a linear upward trend in the proportion of palmitic acid within the total lipid fraction, rising from 15.7% at day 0 to 17.8% by day 7 of exposure. However, the differences between time points were not statistically significant (*p* = 0.294 and 0.265, respectively).

In the transgenic lines, the change in palmitic acid content followed a different pattern. Specifically, in the codA lines, a decrease or stabilization of the 16:0 level was observed on day 2 (e.g., in CodA25, it dropped from 16.8% to 13.5%, representing a significant difference from day 0, *p* = 0.020). By day 7, however, a pronounced compensatory increase occurred, reaching 18.2% in CodA25 (difference from day 0 not significant) and 18.1% in CodA8 (difference significant, *p* = 0.012).

**Figure 9:**
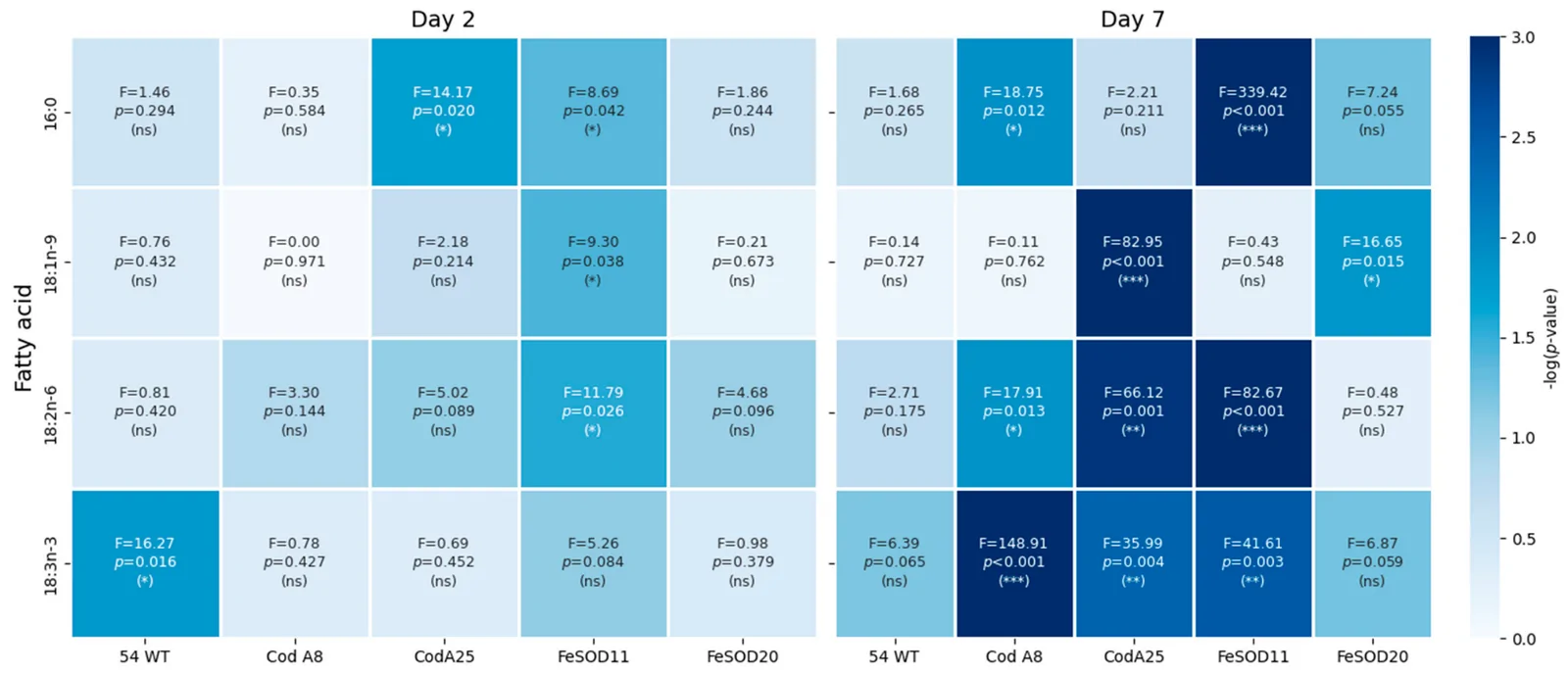
ANOVA results testing for significant differences in the content of major fatty acids within total lipids based on the duration of NaCl exposure (pairwise comparison with day 0). Statistical significance of differences relative to day 0 (*n* = 3): ns—*p* > 0.05, *—*p* < 0.05, **—*p* < 0.01, ***—*p* < 0.001.

**Figure 10:**
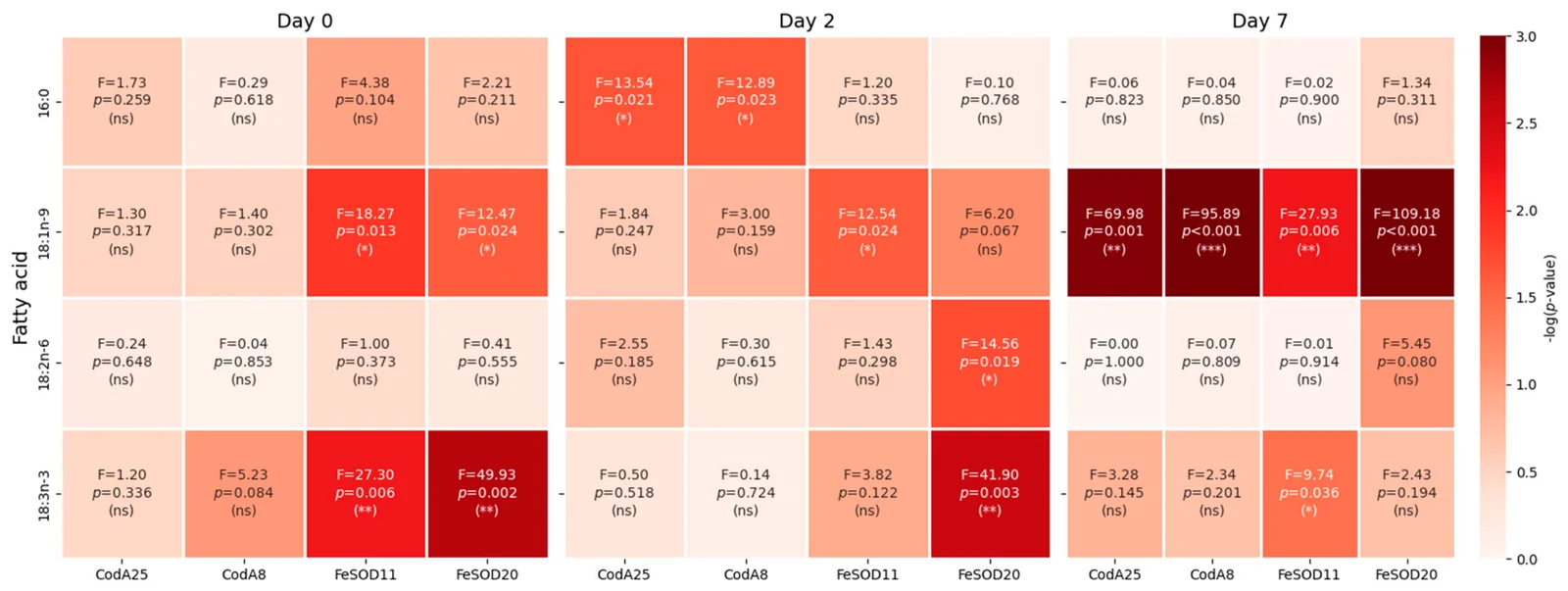
ANOVA results testing for significant differences in the content of major fatty acids within total lipids based on the duration of NaCl exposure. Pairwise comparisons were made against the wild type (WT54) and day 0. Statistical significance (*n* = 3): ns—*p* > 0.05, *—*p* < 0.05, **—*p* < 0.01, ***—*p* < 0.001.

**Figure 11:**
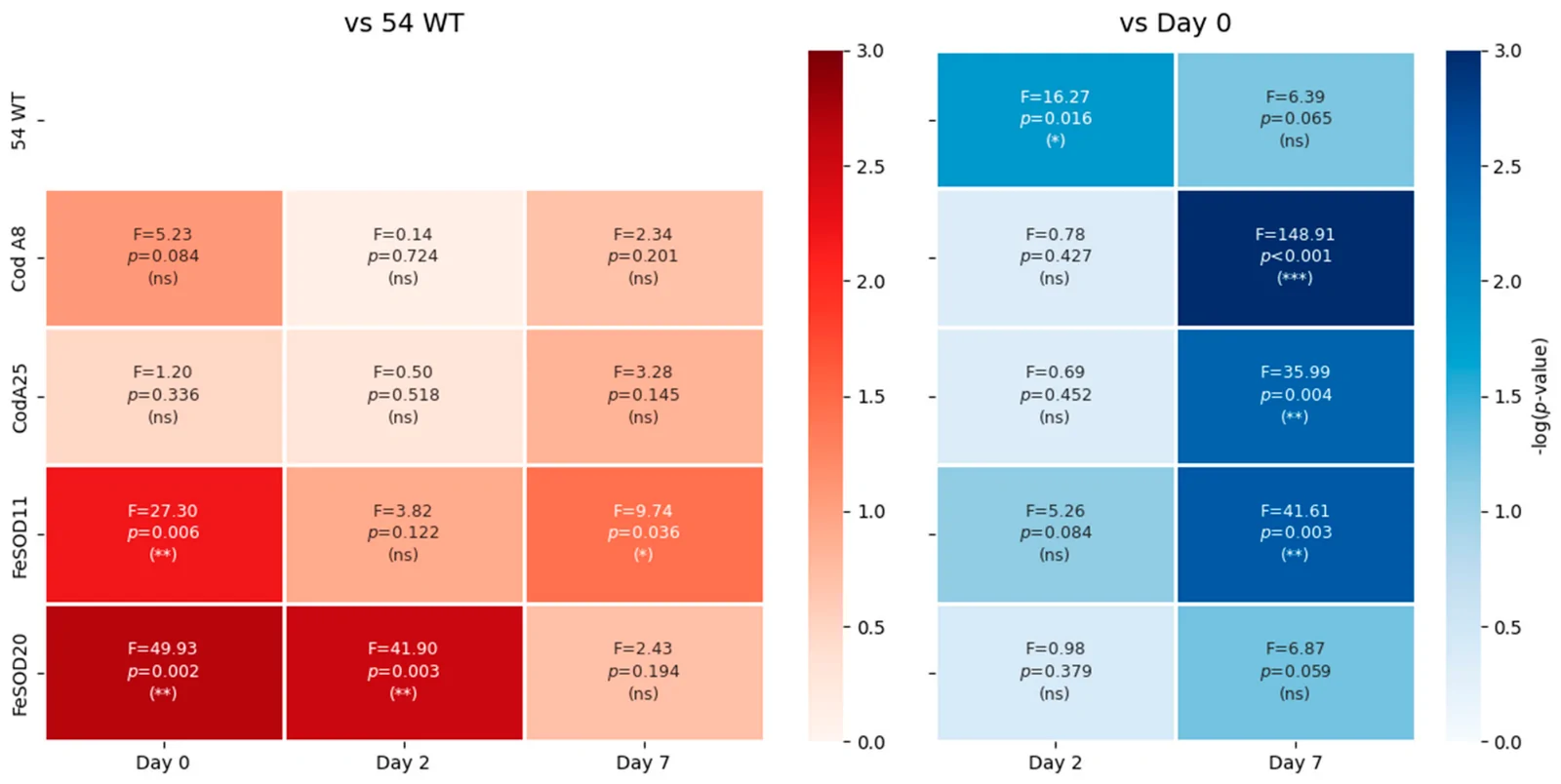
ANOVA results testing for significant differences in α-linolenic acid content within total lipids based on stress exposure duration across experimental variants. Pairwise comparisons were conducted between transgenic lines and the wild type (WT54), as well as relative to day 0 of the experiment. Statistical significance (*n* = 3): ns—*p* > 0.05, *—*p* < 0.05, **—*p* < 0.01, ***—*p* < 0.001.

In the transgenic lines (particularly FeSOD11 and FeSOD20), the initial level of oleic acid was significantly higher (20.6% and 19.4%, respectively) than in the wild type (14.7%). The exceptions were lines CodA8 and CodA25, where the differences from the wild type were not statistically significant. By day 7, a significant increase in the relative abundance of oleic acid within the total fatty acid composition was observed across all lines; this effect was particularly pronounced in lines FeSOD20 and CodA8 (*p* < 0.001; F = 109.18 and F = 95.89, respectively) (Table 1, Figure 9, Figure 10 and Figure 11).

Of particular interest is the change in the pool of polyunsaturated α-linolenic acid (18:3), which governs membrane fluidity. On day 0, the 18:3 content in lines CodA8 and CodA25 did not differ significantly from that of the wild type (WT54), whereas in lines FeSOD20 and FeSOD11, it was significantly lower (*p* = 0.002 and *p* = 0.006, respectively).

On day 2 of stress, all examined transgenic lines showed a statistically non-significant trend toward a slight increase in the proportion of 18:3 within total lipids compared to WT54 (Figure 11). However, by day 7, the transgenic lines CodA8, CodA25, and FeSOD11 exhibited a sharp, significant drop in 18:3 content (to 37.4% in CodA25 and 33.8% in FeSOD11) relative to day 0. This decline was more pronounced than in the wild-type line (which showed a statistically insignificant decrease to 41.4%) and the transgenic line FeSOD20 (where the reduction to 37.4% was also statistically insignificant compared to day 0). When compared directly to theWT54 line, a statistically significant decrease in 18:3 acid to 33.8% after 7 days of exposure was observed only in the FeSOD11 line (*p* = 0.036, F = 9.74).

Thus, it can be concluded that the expression of heterologous genes modifies the lipid response to stress. Transgenic plants exhibit a more pronounced biphasic response. The initial maintenance of high membrane unsaturation (day 2) is followed by a sharp shift toward the synthesis of signaling molecules. This shift is indirectly indicated by the consumption of 18:3n-3, a precursor for jasmonic acid (a key stress hormone that triggers a cascade of defense reactions). Furthermore, an increase in membrane rigidity is indirectly evidenced by the rise in the proportion of saturated fatty acids—predominantly palmitic acid—by day 7 of exposure.

### 2.9. Composition of Volatile Organic Compounds After Salt Stress Impact

Salt stress induced changes in the composition of volatile organic compounds in the leaves of the studied transgenic forms that differed from the response observed in the parental form (Table 2).

Table 2 illustrates the effect of salinity on the secondary metabolism of mint, specifically regarding the synthesis of essential oil components, which are of primary economic and pharmacological importance.

**Figure 12:**
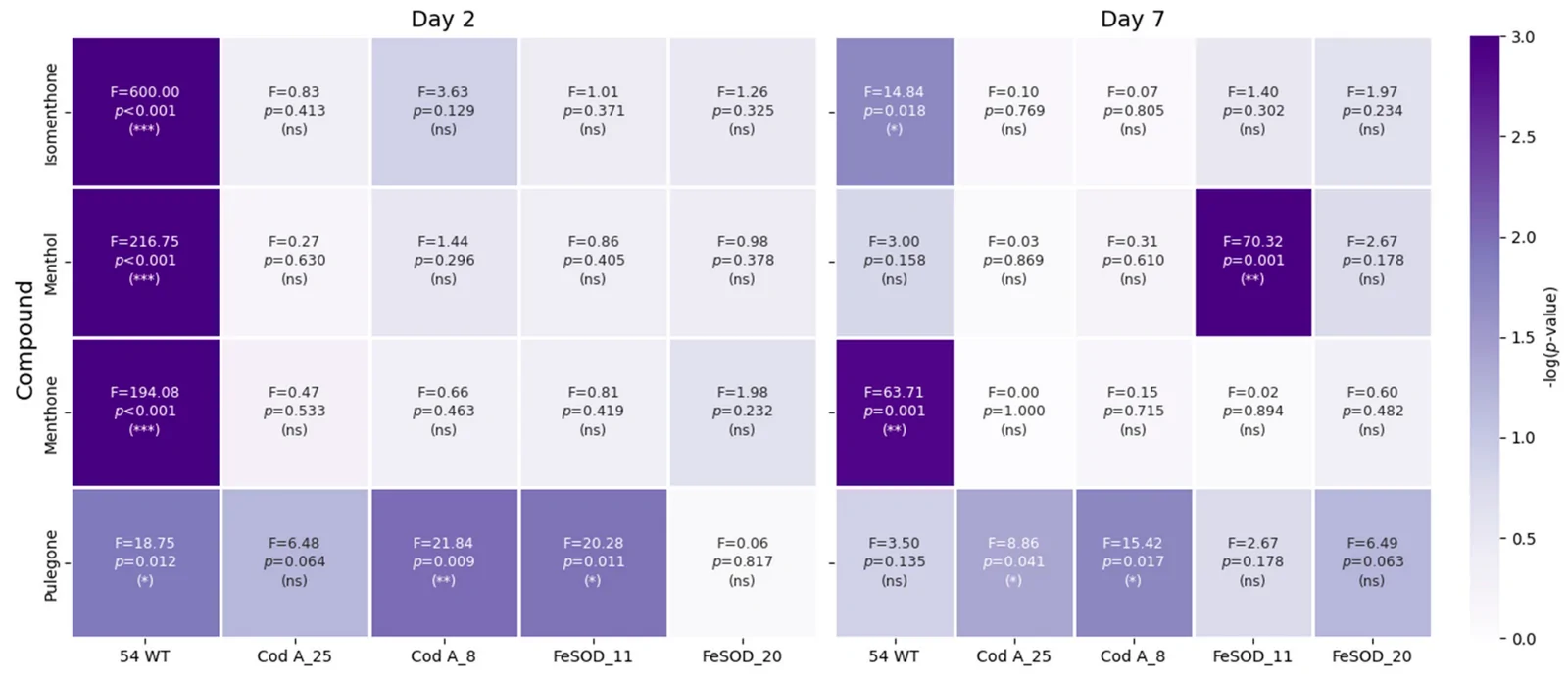
ANOVA results for the quantity (mg/kg fresh weight) of target volatile mint metabolites across experimental lines. Statistical significance of differences relative to day 0 (*n* = 3): ns—*p* > 0.05, *—*p* < 0.05, **—*p* < 0.01, ***—*p* < 0.001.

In the wild type, salt stress had a detrimental effect on menthol accumulation (Figure 12). Menthol content steadily declined from 0.93 mg/kg (in the control) to 0.61 mg/kg by day 7; however, a statistically significant difference in the absolute quantity of this metabolite was observed only on day 2 (Fig. D, *p* < 0.001). By day 7, despite a continuing downward trend in the absolute amount of menthol, the difference compared to day 0 was not statistically significant (*p* = 0.158) due to a high coefficient of variation resulting from a large standard deviation. In contrast to the wild type, all transgenic lines demonstrated high stability in menthol content on day 2 of salt stress exposure (no statistically significant differences relative to day 0 were found in any line). This effect persisted through seven days; notably, the FeSOD11 line even showed a statistically significant increase in menthol quantity compared to day 0 (from 0.81 to 1.12 mg/kg fresh weight, *p* < 0.001).

**Figure 13:**
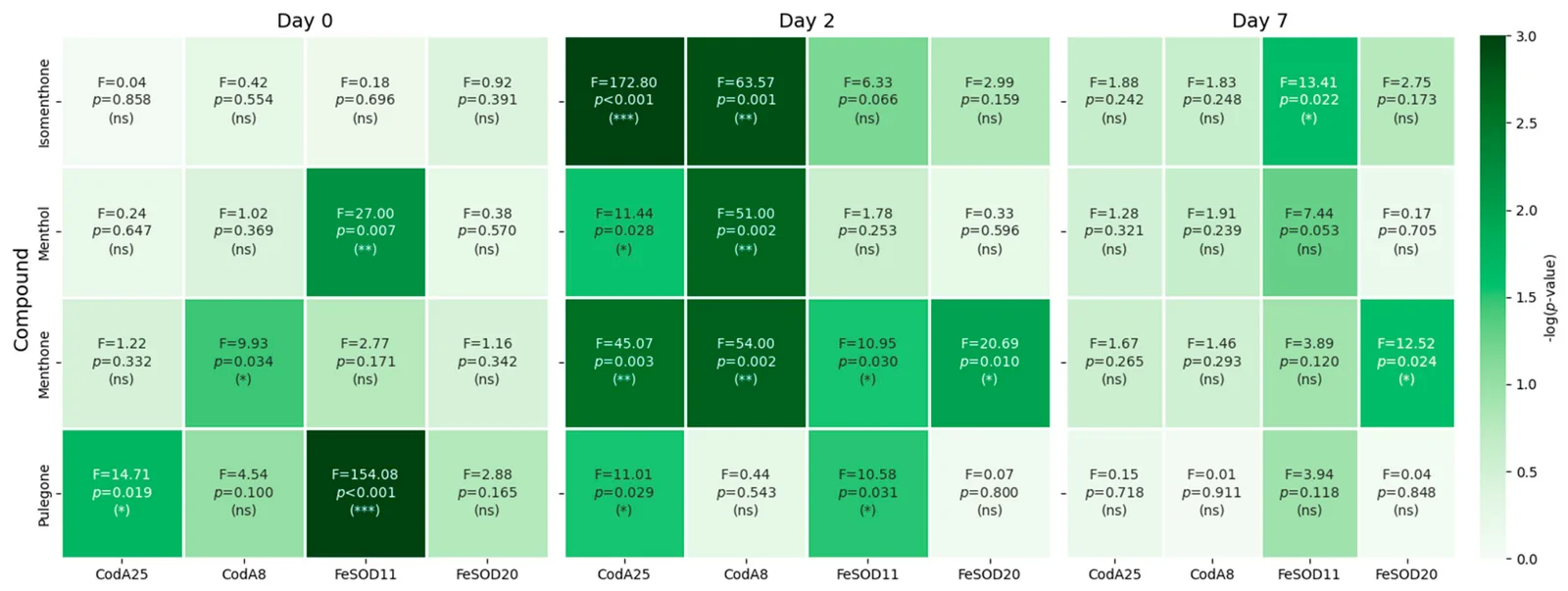
ANOVA results for the quantity (mg/kg fresh weight) of target volatile mint metabolites across experimental lines. Pairwise comparisons were made against the wild type (WT54) and day 0. Statistical significance (*n* = 3): ns—*p* > 0.05, *—*p* < 0.05, **—*p* < 0.01, ***—*p* < 0.001.

Similar trends were observed for the content of menthol’s metabolic precursors, menthone and isomenthone. In the wild-type plants, a sharp, statistically significant decrease in the levels of menthone (from 0.68 to 0.30 mg/kg; *p* < 0.001, F = 194.08) and isomenthone (from 0.56 to 0.27 mg/kg; *p* < 0.001, F = 600.0) was observed on the second day. Conversely, in all transgenic lines, the content of these metabolites remained stable or showed only a slight, statistically insignificant decline. This trend persisted throughout the 7-day experiment, indicating that a consistently high level of precursor metabolites was maintained. This suggests that the yield of target metabolites could have been even higher with further maturation of the transgenic mint lines (Figure 13).

The dynamics of the hepatotoxic compound pulegone—an undesirable metabolite—warrant separate consideration (Figure 13). Under normal conditions (day 0), the baseline pulegone level in most transgenic lines was initially lower than in the wild type (1.07 mg/kg). For instance, the content was 0.60 mg/kg in the FeSOD20 line (difference from WT54 not statistically significant, *p* = 0.165), 0.64 mg/kg in the FeSOD11 line (*p* < 0.001), and 0.76 mg/kg in the CodA25 line (statistically significant difference, *p* = 0.019). Under stress, pulegone levels increased by day 7, though not to a statistically significant degree in any of the lines (*p* = 0.118–0.848). Nevertheless, the fact that these transgenic plants initially exhibited lower levels of this toxin represents a highly beneficial side effect of the genetic transformation.

These results indicate that the introduction of the *codA* and *FeSOD* genes exert a strong protective effect on the secondary metabolism of mint. While salt stress suppressed essential oil synthesis and led to the degradation of valuable menthol in the wild type, the transgenic lines maintained high biosynthetic pathway activity, ensuring a stable or even increased yield of menthol and its precursors. These findings demonstrate that genetic modification protects the cellular biosynthetic machinery, enabling the plants to not only maintain but also enhance the synthesis of the target product under stress conditions.

It can be seen that the composition of essential fatty acids and their content in total lipids of peppermint leaves remained virtually constant across all samples studied in both wild-type (line 54WT) and transgenic lines. For example, in line 54, no statistically significant differences were found not only in the content of individual fatty acids but also in the integral parameter—unsaturation index (F = 0.4903, *p* = 0.3968). However, in variant 54, compared with the wild-type, the transgenic lines were characterized by a significantly reduced unsaturation index (F = 6.1736, *p* = 0.0007). This was due to a significant increase in linoleic acid content (F = 34.4046, *p* = 0.0002) and a decrease in the amount of α-linolenic acid in total lipids of transgenic lines compared to the control (F = 8.8346, *p* = 0.0011). Apparently, genetic transformation led to a decrease in the activity of desaturases responsible for the biosynthesis of linolenic acid from linoleic acid.

To assess the effect of heterologous gene expression on the biochemistry of mint plants, the essential oil composition of the original genotype and *FeSOD*-transgenic lines was evaluated. Although these in vitro-cultured plants were small, it was important to establish that no fundamental metabolic shift or change in the composition of the main essential oil components occurred (Figure S4, Table 1). A characteristic ratio of various volatile organic compounds can be noted, the most significant of it is menthol, the content of which remained stable, while the content of pulegone was slightly reduced in the transgenic lines. The mass and diversity of minor volatile organic compounds were also increased (Table S1).

### 2.10. Spectral Characteristics of Transgenic and Wild-Type Mint Lines In Vitro Culture After Salt Stress Impact

Detailed pairwise comparisons and the spectral reflectance characteristics of the studied samples—analyzed via hyperspectral imaging and the calculation of optimal indices—revealed distinct differences in the spectral reflectance profiles of mint samples examined in vitro (Figures S8–S10). It was determined that glass test tubes do not hinder the identification of differences in leaf reflectance, allowing for the assessment of vegetation indices, such as NDVI and others (Figure S10). The differences identified through pairwise comparisons make it possible to detect the effects of damage and alterations in pigmentation (Figures S9 and S10), which can be leveraged in comparative experiments.

### 2.11. The Structure of Epidermal Tissue Cells, Essential Oil Trichomes and Stomata

**Figure 14:**
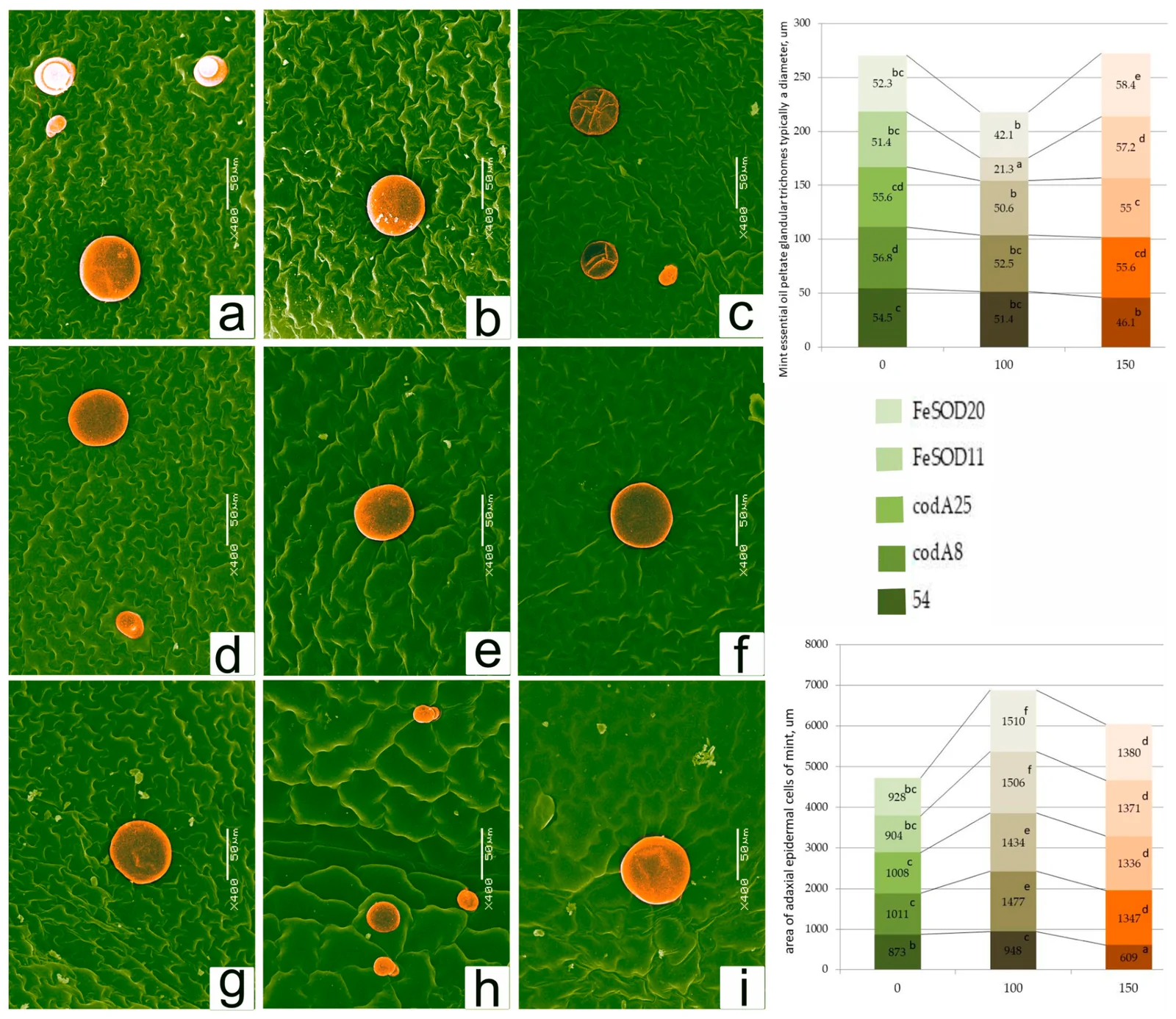
The surface structure of the adaxial side of the leaf and morphometric characteristics of the epidermis (green color) and essential oil-containing trichomes (orange color) in wild-type mint line plants (**a**–**c**) and *FeSOD*- (**d**–**f**) and *codA*-transgenic (**g**–**i**) lines when cultivated in vitro on MS medium (**a**,**d**,**g**); 100 mM NaCl (**b**,**e**,**h**) and 150 mM NaCl (**c**,**f**,**i**). In the legend, the corresponding accessions are indicated by a staining intensity similar to that observed under conditions without NaCl treatment.

The adaxial surface structure of mint leaves exposed to salt underwent characteristic changes in sensitive patterns such as epidermal cell size and trichomes (Figure 14). In plants of the original genotype, epidermal cell shape changed at 100 mM NaCl, apparently due to a disruption in turgor. Cell square remained stable, while the diameter of the peltate glandular trichomes decreased. Exposure to 150 mM NaCl significantly reduced epidermal cell area and the diameter of peltate glandular trichomes. *FeSOD*- and *codA*- transgenic lines, when grown on MS medium without salt influence, showed a tendency toward increased epidermal cell area. However, the diameter of essential oil trichomes was somewhat lower in *FeSOD*-transgenic plants, while *codA*-transgenic lines remained similar to or slightly higher than the control. *FeSOD*- and *codA*-transgenic lines exposed to 100 mM NaCl exhibited a significant increase in epidermal cell area and a decrease in the diameter of peltate glandular trichomes. At 150 mM NaCl, an even greater increase in epidermal cell area was observed, associated with uniform growth, more pronounced in *codA*-transgenic lines. Trichome diameter, however, was consistent with that without salt treatment.

**Figure 15:**
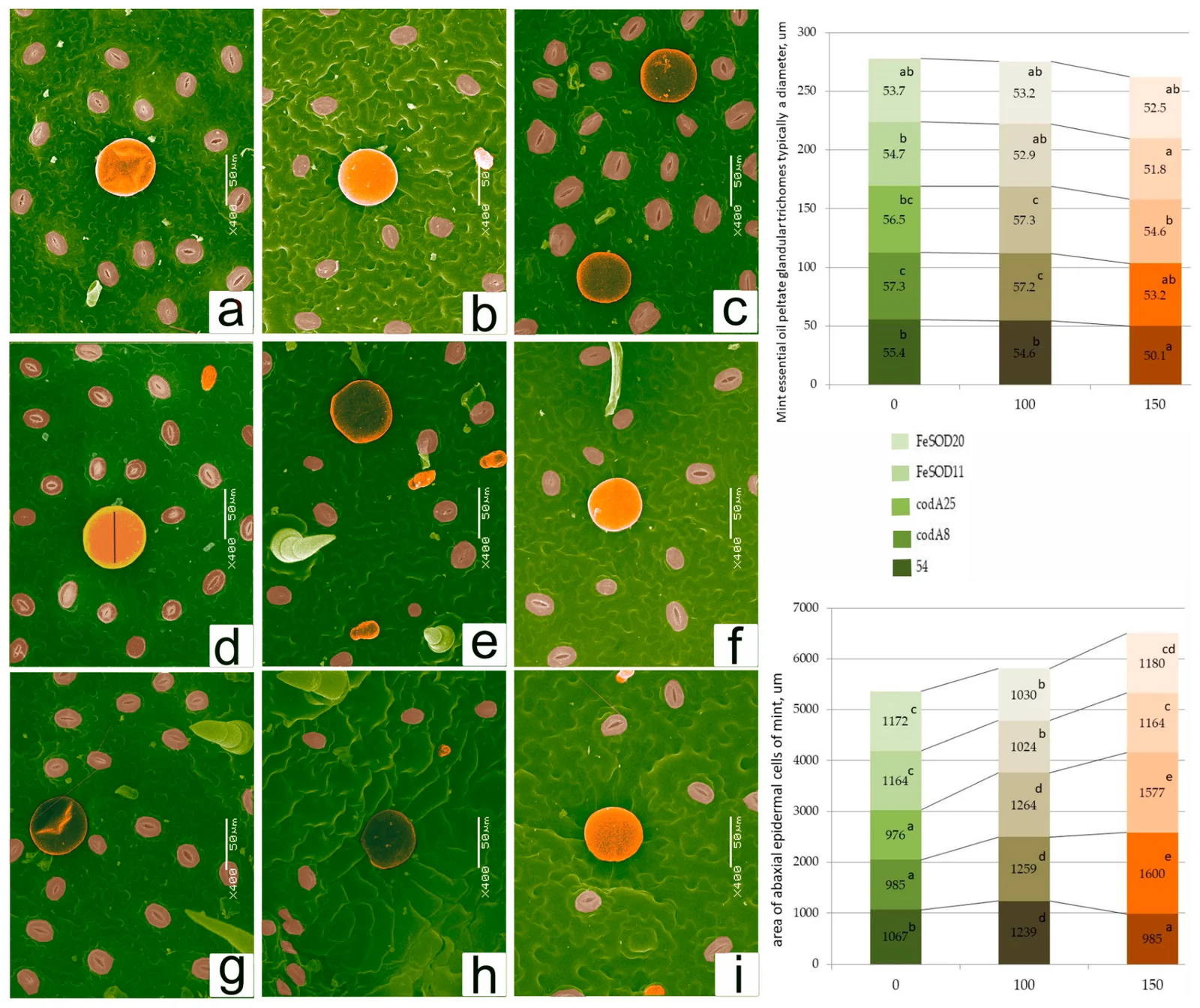
Abaxial leaf surface structure and morphometric characteristics of the epidermis (green color), stomatal density (white-orange), and essential oil-containing trichomes (grey-orange) in wild-type mint plants (**a**–**c**) and *FeSOD*- (**d**–**f**) and *codA*-transgenic (**g**–**i**) lines during in vitro cultivation on MS medium (**a**,**d**,**g**); and with the addition of 100 mM NaCl (**b**,**e**,**h**) and 150 mM NaCl (**c**,**f**,**i**). In the legend, the corresponding accessions are indicated by a staining intensity similar to that observed under conditions without NaCl treatment.

The effects of *FeSOD* and *codA* resulted in an increase in the area of epidermal cells on the abaxial side of mint leaves compared to the wild-type genotype, while the effect of *FeSOD* and *codA* expression on the size of peltate glandular trichomes was less manifested (Figure 15). Salt exposure caused an increase in cell area in the wild-type genotype at 100 mM NaCl, but 150 mM NaCl inhibited cell growth, and cell growth remained smaller than in the control when cultured on MS. *FeSOD*-transgenic plants responded to exposure to 100 mM NaCl with an increase in cell area, but at 150 mM NaCl the cell area was not increased. Plants expressing *codA* exhibited a significant increase in epidermal cell area when exposed to both 100 mM NaCl and 150 mM NaCl, more manifested at 150 mM NaCl. The diameter of peltate glandular trichomes was significantly reduced only when exposed to 150 mM NaCl. At the same time, the trichome diameter in *FeSOD*- transgenic plants was slightly smaller than that of the parent line and the line overexpressing *codA*. The effect of NaCl on stomatal density on the abaxial side of leaves was reflected by a decrease in their number at 100 mM NaCl. At 150 mM NaCl, stomatal density in the parent line increased relative to optimal conditions (Figure S7). In transgenic plants, a slight increase in this parameter was observed only in *FeSOD*-transgenic lines, while in *codA*-transgenic lines, the number was reduced by two and a half times compared to the parent line. These changes correlated with the increase in epidermal cell size noted in Figure 15.

## 3. Discussion

Salinity can cause critical physiological and biochemical metabolic disturbances in *Mentha* species. It has now been established that peppermint is very sensitive to soil salinity, since already at 75 mM NaCl (which, according to the classification more accepted by agronomists, is 0.3%), plant growth, increase in biomass and production of essential oil are blocked [23]. In Moroccan mint, at a lower concentration of 50 mM NaCl, inhibition of the ability to vegetative reproduce (which is key for this crop due to problems associated with the diversity in ploidy of many valuable genotypes and the difficulty of ensuring sterility during reproduction [27]) was reliably established by blocking the formation of adventitious roots [28]. As in most glycophytes, an increase in the level of Na^+^ and Cl^−^ ions in the rhizosphere not only affects the osmotic potential of the substrate but also directly interferes with water absorption by the roots, leading to a decrease in leaf cell hydration, a drop in stomatal conductance, and a decrease in chlorophyll concentration [29]. Increased ROS production disrupts compartmentalization due to damage to the lipid composition of membranes [30]. At the same time, this is accompanied by a decrease in chlorophyll content and the K^+^/Na^+^ ratio, further inhibiting photosynthesis, leading to a consequent reduction in plant growth, yield, and the production of valuable biologically active compounds [31,32,33].

The use of *Agrobacterium*-mediated transformation to produce transgenic peppermint plants is currently an effective technology for targeted genetic modification [34,35]. One of the main problems is the selection of the most suitable explant with a high degree of regeneration, for which stem or leaf fragments are usually used [36,37]. In this case, for the selected peppermint sample, leaf blades showed the greatest efficiency of regeneration (Figure S1). As a result of *Agrobacterium*-mediated transformation, the genetic constructs were successfully inserted into the genome of recipient peppermint plants, after which, as expected, expression of the target genes in individual lines was confirmed. Two lines carrying the choline oxidase gene, which enables the synthesis of glycine betaine from choline, as previously demonstrated for tobacco and other plants, were selected for further studies [24,38,39]. The application of this gene, obtained from *Arthrobacter globiformis*, allows both to increase the content of this effective osmotic agent and to regulate oxidative stress [40]. Lines that did not have obvious differences in habit and color were selected to evaluate their resistance to simulated salt stress, which is one of the most studied and relatively easily reproducible in vitro and in vivo conditions [41,42,43].

In the present study, the performance of a Fe-superoxide dismutase transgene, which has previously demonstrated efficacy in protecting against salt-induced oxidative damage in tobacco, was also assessed [25]. In this case, a repetition of the premodulating effect achieved by creating internal stress was expected to enhance the sensitivity and effectiveness of the induction of defense systems when plants encounter stressful influences [18,44]. This is important for mint, which, as a glycophyte, rarely experiences salinity of varying degrees. This means that this species, potentially sensitive and extremely unstable to salinity, can be grown in regions where irrigation can cause secondary salinization [45].

Two lines each with high expression levels of the *FeSOD* (FeSOD11 and FeSOD20) and *codA* (CodA8 and CodA25) genes were selected for subsequent clonal micropropagation. These lines had no morpho-physiological differences from the original genotype and were not visually distinct in growth performance, size, or leaf blades. In order to evaluate possible physiological differences between the obtained *FeSOD* and *codA* transgenic lines, a protocol for in vitro clonal propagation was developed [37]. The obtained transgenic lines were evaluated for a number of physiological parameters, such as biomass accumulation, the amount of photosynthetic pigments and carotenoids, electrolyte leakage, proline and MDA content, lipid composition and essential oil content (Figure 1, Figure 2, Figure 3, Figure 4 and Figure 5 and Figures S1–S4). Although no visual differences or changes in growth were observed in transgenic lines compared to the wild type when cultured on MS medium (Figure S1), a number of physiological parameters were significantly altered, confirming the expected modifications in the physiological metabolism of the studied lines. For example, seedling water content, assessed by differences in fresh and dry plant biomass, was significantly lower, while differences in fresh weight were insignificant (Figure 1). Such effects may indicate that cell growth and division differ from what occurs in plant tissues without stress [45]. At the same time, a decrease in the pool of photosynthetic pigments and carotenoids was noted in both *FeSOD*-transgenic lines and *codA*-transgenic lines (Figure 2 and Figure 3), which is a characteristic reaction of plants to the adverse effects of the presence of NaCl in the environment [9,45]. Differences in proline and MDA content were observed both between lines with different transgenes and between them and the original genotype. In some cases, an increase was noted in one line and a decrease in another. One of the *codA*-transgenic lines showed no differences from the original genotype. These differences are possibly related to a metabolic imbalance caused by altered proline biogenesis, which has been noted previously [46]. A change in the MDA pool and some changes in the composition of lipids and essential oil were also observed, indicating a non-damaging but possible influence of internal conversions of oxidative processes and the osmotic state under the influence of heterologous genes (Figure S5).

NaCl significantly reduced the growth of fresh biomass, associated with a significant reduction in growth in both the original genotype and the transgenic mint lines (Figure 1). However, the biomass and condition of the transgenic plants were significantly improved at both 100 mM NaCl and 150 mM NaCl. This suggests that nonspecific defenses induced by superoxide dismutase and choline oxidase can reduce risks and increase plant survival under conditions of temporary increased salinity associated with drought. Such encouraging results may be of great value for mint plants, which are typically intolerant to 75 mM NaCl [47]. However, the *FeSOD* and *codA* genes used in transgenosis, whose functioning had previously shown effectiveness on facultative glycophytes, could not significantly prevent the decline in biomass growth at high salt concentrations [48,49,50]. Salinity caused a significant slowdown in dry biomass gain, associated with growth inhibition in both the original genotype and the transgenic mint lines (Figure 1). This effect was expected for such a sensitive glycophyte, and the *FeSOD-* and *codA-* transgenic lines used in the study could not have significantly affected the parameters associated with the functioning of the transporter system regulating the transport and accumulation of ions. This is characteristic of plants such as tomatoes, which are facultative halophytes and capable of accumulating ions in their cells at relatively high concentrations [51,52,53]. It can be noted that a slight tendency toward greater biomass growth was noted for the transgenic lines studied, which was significant at 100 mM NaCl (CodA8) and 150 mM NaCl (FeSOD20). However, the effect was not as noticeable as that observed earlier in moderately salt-tolerant glycophytes such as tomato and tobacco [25,48]. Apparently, this result is caused by the absence of mechanisms of non-specific protection against salinity, capable of inducing changes in membrane ion transport complexes [54,55].

Under optimal conditions, chlorophyll a content was expectedly reduced in transgenic plants, which was caused by the fact that the products of superoxide dismutase and choline oxidase activity directly or indirectly caused premodulation of oxidative stress, as we showed previously [39]. However, when transgenic plants were faced with salinity, which is characterized by the combined effects of oxidative, osmotic, and toxic stress [56,57], they demonstrated greater protection and, accordingly, higher performance (Figure 2 and Figure 3). At the same time, the carotenoid pool was stable and showed high values, close to the norm for transgenic lines, but did not reach the value characteristic of the original genotype (Figure 8). Thus, the photosynthetic system of transgenic plants expressing the *FeSOD* and *codA* genes demonstrated high stability and resistance, which can be judged by the increased pool of chlorophylls a and b relative to the original variant (Figure 2 and Figure 3). Despite the significant decrease in these parameters, they were higher than those of wild-type plants and, therefore, retained a high potential to quickly compensate for photosynthetic activity and ensure growth after a decrease in salt concentration [58].

The proline content of transgenic plants, when grown under optimal conditions without salinity, was higher than that of the wild type or corresponded to the values demonstrated by the original line, with the exception of the FeSOD20 line, whose values were reduced under optimal conditions and at 100 mM NaCl (Figure 4). This may have been caused by differences in gene expression associated with one of the factors influencing the formation of differences, such as the site of transgene insertion, or damage to another gene affecting the metabolic chain [59]. Long-term exposure to 100 mM NaCl caused a decrease in proline levels in transgenic lines, while in the wild type, an increase was observed at both 100 mM and 150 mM NaCl. This indicated increased sensitivity of this parameter specifically in the transgenic lines. At higher concentrations, either stable levels were observed or an increase to baseline values was observed, including for FeSOD20. This response can be explained by the fact that mint plants in this case are unable to effectively utilize the strategy of increasing proline synthesis to protect their cells. A decrease or lack of significant changes in cellular proline levels during prolonged salinization may indicate a lack or impairment of the plant’s adaptive mechanisms, as well as depletion of energy resources [60].

One of the indicators of the state of membrane systems is lipid peroxidation, which remained relatively stable and low in transgenic lines, but increased significantly in the wild type at 150 mM, when plant growth practically stopped (Figure 5). Thus, the MDA indicator indicated that high salinity significantly damaged the membrane system of mint plants, which is typical for this type of stress [61]. A noticeable reduction in significant damage in transgenic plants demonstrates the feasibility of using this approach, as the response was generally consistent with the effect previously established on *codA*-transgenic tobacco plants under 200 mM NaCl impact [47].

Reactive oxygen species, including H_2_O_2_, trigger stress responses, acting as signaling agents. It was observed that while peroxide levels in *codA*-transgenic plants were 1.5 times higher in the absence of salinity, hydrogen peroxide levels in transgenic plants carrying the Fe-dependent superoxide dismutase gene were 2–3 times higher in the FeSOD20 and FeSOD11 lines, confirming the more significant role of superoxide dismutase in peroxide production, although both products affect peroxide levels [39]. When plants are exposed to high salt concentrations, a significant increase in peroxide is observed in non-transgenic plants. Conversely, peroxide levels are elevated, but relatively stable, in transgenic lines. Consequently, the level of peroxide neutralization in transgenic lines overexpressing superoxide dismutase is even lower than in the same lines grown in a sodium chloride-free environment. Thus, the protective function of neutralizing reactive oxygen species is quite significant here (Figure 6). This confirms the effectiveness of such a strategy to protect plant cells from damage, as in other studies where peroxide and glycine betaine spray treatments were used [62,63,64,65].

In vitro culture, like any model system, has some differences from natural morphology, which is associated with the characteristics of closed systems that change normal gas exchange and air humidity due to limited volume and lack of air flow [66,67]. Leaves of plants in vitro culture have a different number of stomata, altered mesophyll and epidermal cells, and are likely characterized by greater uniformity of organs across tiers due to greater availability of light fluxes and stability of microconditions [68,69]. Thus, it was observed that the diameter of the peltate glandular trichomes (Figure 14 and Figure 15) was quite stable, while usually in mint there may be differences in this parameter in different parts of the plant, especially under stress [70]. Trichomes and stomata are also quite sensitive to in vitro conditions [36,71]. Morphological differences between the upper and lower epidermis of *FeSOD*- and *codA*-transgenic plants under in vitro culture conditions in the absence of salt exposure demonstrated that, although there were no visually noticeable differences between the microcloned plants, there were differences in the cell size of the upper and lower epidermis (Figure 14 and Figure 15), the size of the peltate glandular trichomes (changed) and the number of stomata on the abaxial side of the leaf (Figure S7). A decrease in the density of stomata against the background of an increase in the area of cells indicated the completion of the processes of division and morphogenesis in leaf tissues and the end of the formation of stomata, as an important organ providing water and gas exchange, which was observed at 100 mM NaCl (Figure 15 and Figure S7). On the other hand, stomatal cell size decreased, indicating a decrease in respiration and transpiration, and therefore in the efficiency of photosynthesis. At 150 mM NaCl, stomata were larger and their density increased (Figure 15). In the non-transgenic (wild type) line, the epidermal cells were significantly smaller, but contained a large number of stomata, which indicated the cessation of growth and the preservation of the leaf in the state in which it was before being placed in saline conditions, since no plant growth was observed under these conditions (Figure 15). Obviously, the morphological changes associated with epidermal cell enlargement and the formation of new stomata observed in transgenic plants cannot be detected in this situation. Comparing the leaf morphology of *FeSOD*- and *codA*-transgenic plants, these plants demonstrated greater stability, as they did not exhibit the proliferation and unevenness of epidermal cells observed in *codA* lines (Figure 15f,i).

Alterations in lipid composition help plants maintain cellular stability, regulate osmotic pressure, and protect the photosynthetic apparatus from the toxic effects of sodium and chloride ions [72,73]. The sensitivity associated with α-linolenic acid appears to be linked to the fact that the gene products—enzymes—possess a signal sequence that targets them to the chloroplast, thereby rendering this compartment more vulnerable [72,73]. High concentrations of polyunsaturated fatty acids (especially 18:3 and 18:6) are critical for chloroplast thylakoid membranes. An increase in their content indicates the maintenance of optimal photosystem II (PSII) function under salinity stress, as well as the protection of chloroplasts against photoinhibition (light-induced damage occurring under stress conditions) [74]. It is also known that free and lipid-bound fatty acids serve as precursors for the synthesis of various protective molecules. For instance, linolenic acid (18:3) is a precursor for jasmonic acid—a crucial stress hormone that triggers the expression of salt-tolerance genes and activates the plant’s antioxidant system [75]. It is possible that the salinity tolerance of transgenic mint plants is indirectly linked to this activation and this protective system.

Thus, it can be concluded that the chosen protection strategy using heterologous enzymes that indirectly affect the oxidative potential of plants is capable of preventing complete inhibition of their development and gives hope that in the case of a decrease in salinity levels, for example, during irrigation or during co-cultivation with salt-absorbing crops, the modified mint plants will be quite productive. We also believe that spraying mint with the enzyme products used in the study, namely glycine betaine and hydrogen peroxide, could likely produce similar effects [63,76]; however, they would not have a prolonged effect and could require increased costs for farmers.

## 4. Materials and Methods

### 4.1. Plant Material

*Mentha × piperita* L. cv. Mitcham was provided by the essential oil crop collection of the N.V. Tsitsin Main Botanical Garden. *Mentha × piperita* L. (Mitchamx peppermint) grows up to 80 cm tall, has a compact bush, and erect branches. The stem is anthocyanin-colored and glabrous. The leaves are dark green, lanceolate, with sharply toothed edges and glabrous. The dark purple flowers are collected in spike-shaped inflorescences. The plants are mostly sterile due to underdeveloped anthers, so this mint species is propagated only vegetatively by rhizomes. Mitcham mint (or Mitcham) is a variety of peppermint (*Mentha × piperita*) developed in Britain, through an accidental hybrid between spearmint and water mint. It is characterized by a strong aroma and high menthol content. Mint plants were aseptically maintained and multiplicated in glass tubes.

### 4.2. Genetic Transformation

For genetic transformation, previously created pBI-based constructs containing the target genes *Fe-SOD* from *Arabidopsis thaliana* and *codA* from the soil bacterium *Arthrobacter globiformis*, encoding Fe-dependent superoxide dismutase and choline oxidase, respectively, were used (Figure S2) [77]. Finally, binary vectors were transferred into *Agrobacterium tumefaciens* AGL0 using electroporation. Transgenic mint plants were obtained using transformation by means of co-cultivation with *Agrobacterium tumefaciens*. Stem segments and basal parts of leaves were used as explants. After co-cultivation with *A. tumefaciens*, the explants were grown on MS agar medium supplemented with 30 g L^−1^ sucrose, 2 mg L^−1^ trans-zeatin, and 0.2 mg L^−1^ NAA. Developing shoots were transferred to glass tubes containing MS agar medium, where they rooted and multiplied in vitro. Tubes were placed in a climate chamber and cultured at 10,000 lux, 22–24 °C, a 16 h photoperiod, and 75% humidity.

### 4.3. Genomic DNA and Total RNA Isolation of Wild-Type and Transgenic Lineages

Genomic DNA was extracted from all mint plants by standard protocol and total RNA isolated through hot phenol procedure [78]. The purified RNA and DNA were analyzed on a 1% non-denaturing agarose gel containing 0.7% ethidium bromide (EtBr). To purify RNA samples from genomic DNA impurities were used DNAse I (Thermo Fisher Scientific, Waltham, MA, USA). First strand cDNA synthesis was performed with MMLV RT kit (Evrogen, Moscow, Russia), oligo (dT) primer according to the manufacturer protocol. The primers used for amplifying the *FeSOD* gene were 5′GCAGTCAGTATTAGGCTTA3′ and 5′CCATCAACTACATAAACAACA3′, and the primers for the *codA* gene were 5′CGCCAACTTCTTCCAGATCAA3′, and 5′GGGTGTTCATGTCGAACG3′. The PCR mixtures were incubated under the following conditions: one cycle of 95 °C for 5 min followed by 45 cycles of 95 °C for 30 s, 56–58 °C for 15 s, and 72 °C for 20 s. Between 30 and 100 ng of the template was added to the sample. The final PCR products were analyzed on a 1% non-denaturing agarose gel (Figure S1).

### 4.4. qPCR in Transgenic Plants

The Quantitative real-time PCR (qPCR) was obtained by ANK-32 thermocycler (Synthol, Moscow, Russia) under the following conditions: The PCR program consisted of pre-denaturation at 95 °C for 5 min and 45 cycles of the following steps: 95 °C for 20 s, 56–58 °C for 15 s, and 72 °C for 20 s. Between 30 and 100 ng of the template was added to the sample (based on the amount of RNA taken for cDNA synthesis). The OD260/280 of each sample ranged between 1.8 and 2.1, while OD260/230 ranged between 2.17 and 2.41. The final PCR products were analyzed on a 1% non-denaturing agarose gel (Figure S2).

### 4.5. Determination of Sensitivity to Salt Stress

In vitro plants were used for the experiment. These included control non-transgenic plants (cv Mitcham) and randomly selected plants from transgenic lines with confirmed transgene expression (*FeSOD* and *codA*) through molecular analysis. Segments of 4-week-old plants with two opposite leaves were used as explants. Each explant was sterilely transferred to a test tube containing 15 mL of agarized ½ MS medium supplemented with 100 and 150 mM NaCl, respectively. Ten plants were used per replicate for each salinity simulation. The test tubes were placed in standard racks and cultivated in a climate chamber at 10,000 lux, 22–25 °C, and 75% humidity for 50 days. After growth under control and salt-exposed conditions, the selected plants underwent morphometric and biochemical measurements.

### 4.6. Morphometric Characteristics

To analyze the plants, 3 mL of distilled water was added to each test tube, gently shaken, and removed from the agar using a bacteriological loop. To measure the height of the above-ground organs and the length of the roots, the plants were pressed against a ruler. To determine dry and wet weight, five plants were blotted with filter paper, and the roots and green parts of each plant were weighed separately. The remaining five plants were used to determine pigments, proline, MDA, and membrane lipid composition, as well as to fix samples for scanning electron microscopy (TEM). The third pair of leaves from the top was used for pigments and proline determination, the second pair for MDA determination, and the fourth and fifth pairs were used to determine membrane lipid composition.

### 4.7. Determination of Pigments

Extraction of pigments from leaves was carried out with 96% ethanol [79]. The leaves of the plant (0.1 g of fresh weight) were placed in volumetric test tubes and filled with 4 mL of ethanol. To determine chlorophyll, leaf tissue was ground in a mortar with the addition of a small amount of zeolite. The homogenate was centrifuged at 10,000 rpm at 4 °C for 10 min. The absorption of the supernatant was measured at 649, 665 nm on a Genesis 20 spectrophotometer (ThermoSpectronic, Sandy Spring, MD, USA), and the content of pigments was calculated by the following formula:

C_chl a_ = 13.70 E665 − 5.76 E649;

C_chl b_ = 25.80 E649 − 7.60 E665;

C_car_ = (1000E471 × 2.13C_chl a_ − 97.64C_chl b_)/209

A = C·V/1000·W, where C—concentration of pigments, V—extract volume, mL, W—sample weight, g, E—optical density. The pigment content is indicated in μg g^−1^ fresh weight.

### 4.8. Determination of Proline Content

Extraction and determination of free proline were performed according to the method of Bates et al. [80] with minor modifications. A weighed sample of plant material weighing 0.1 g was triturated in liquid nitrogen. The ground material was transferred into test tubes and filled with 4 mL of distilled water. Test tubes with weighed samples were brought to a boil three times, cooling each time. The resulting extract was filtered into measuring tubes. The resulting extract was adjusted to the desired volume (6–7 mL) and then used for analysis. Tubes with 1 mL extract, 1 mL glacial acetic acid, 1 mL ninhydrin reagent (1.25 g ninhydrin, 20 mL 6M H_3_PO_4_, 30 mL CH_3_COOH) were incubated for 1 h in a boiling water bath. Instead of the extract, 1 mL of distilled water was added to the control sample. The optical density of the obtained colored solutions was measured on a PD-303 spectrophotometer (Apel, Saitama, Japan) against the control at a wavelength of 520 nm. The content of proline was calculated using the calibration curve according to the formula:

C = Ek × V/(W × 1000),

where

C—proline concentration, µmol g^−1^ wet weight
E—optical density
k—coefficient calculated from the calibration curve
V—extract volume, mL
W—sample weight, g.

### 4.9. Determination of Lipid Peroxidation

Peroxidation of lipids in plant leaves was analyzed by the content of malondialdehyde (MDA) determined in the reaction with thiobarbituric acid (TBA) [81]. A sample of plant tissue weighing 0.2 g was homogenized in 10 mL of 0.1 mol L^−1^ TRIS-HCl buffer with pH 7.6 containing 0.35 mol L^−1^ NaCl. To 3 mL of the homogenate was added 2 mL of 0.5% TBA in 20% TCA. The resulting mixture was heated at 100 °C for 30 min, quickly cooled, and filtered [82]. The optical density of the filtrates was measured at 532 and 600 nm. The MDA concentration was calculated using the molar extinction coefficient equal to 1.56 × 10^–5^ M^–1^ cm^–1^. The analysis was performed in three repetitions and the concentration was calculated by the formula

C = (E532 − E600) × n/(ε × l × m),

where

C—the concentration of MDA, µmol MDA g^−1^ fresh weight
E—optical density of the solution
n—dilution
ε—Coefficient of molar extinction MDA (156 mM^−1^·cm^−1^)
l—The length of the optical path of the light beam, cm.

### 4.10. Analysis of Fatty Acid Composition of Total Lipids

To obtain fatty acid methyl esters of total lipids from mint leaves, fresh leaves were frozen with liquid nitrogen and ground to powder in a mortar and pestle. 200 mg of homogenized biomass were then weighed into 2 mL glass vials, 200 μL of a solution of margaric acid in 2-propanol (250 μg ml^−1^), 400 μL of ethanol, and 120 μL of a 5M aqueous KOH solution were added. The vials were incubated for 1 h in an Allsheng MK-20 solid-state thermostat (Allsheng Instruments Co., Ltd., Hangzhou, China) at 70 °C, free fatty acids were extracted and were then converted to methyl esters with a 1% solution of sulfuric acid in methanol, which were then analyzed by GC-MS on a 60-column R_T_x -2330 (Restec Corp., Bellefonte, PA, USA) as described previously [83].

### 4.11. Analysis of Volatile Organic Compounds

Fresh mint leaves were frozen in liquid nitrogen and immediately homogenized with a pestle in a porcelain mortar, after which 1 g of biomass was weighed into a 20 mL amber glass vial, 5 mL of dichloromethane was added, than was vortexed and kept in a refrigerator at +4 °C for 2 h, then 3 mL of the extract in dichloromethane was filtered through a 0.45 μm PTFE syringe filter (Agilent Technologies, Santa Clara, CA, USA), 400 μL of a naphthalene solution in dichloromethane (0.0052% *w*/*v*) was added as an internal standard and concentrated to a volume of 250 μL in an argon stream at room temperature. The concentrated extracts (250 μL) were analyzed by gas chromatography–mass spectrometry using an Agilent 7890A GC coupled to an Agilent 5975C MSD (Agilent Technologies, Santa Clara, CA, USA) equipped with a DB-5ms Ultra Inert capillary column (60 m × 0.32 mm i.d., 1.0 μm film thickness). A 1 μL aliquot of each sample was injected in splitless mode at an injector temperature of 275 °C. Helium was used as carrier gas at a constant flow rate of 1.0 mL/min. The oven temperature was programmed as follows: 40 °C (held for 4.7 min), increased to 320 °C at 5.1 °C/min, and held for 7 min. The GC-MS interface was maintained at 280 °C. The mass spectrometer operated in electron ionization mode (70 eV) with a scan range of *m*/*z* 35–450. Compound identification was performed by matching mass spectra against the NIST 2020 library and confirmed by comparison with literature data on mint volatile composition. Peak areas were normalized to the total chromatogram area to express results as relative percentages; the internal standard (naphthalene) was used only for retention time monitoring and was not involved in quantification.

### 4.12. Determination of Electrolyte Leakage and Ion Absorption

To determine the content of ions (Na^+^, K^+^) in the tissues of tomato leaves, the elements were extracted for 30 min at 40 °C with 25 mL of deionized water using an ultrasonic device (Sapphire, Moscow, Russia) from a fresh sample weighing 200 mg. The ultrasound power level was 35 kHz. The samples were cooled, passed through a porous filter (0.45 μm) and used for ions content analysis. Determination of the content of elements in samples was carried out using ion-selective electrodes: using a potassium electrode (ELIT-031); sodium-sodium electrode (ELIS-112); chlorine-chloride electrode (ELIT-261) on an ITAN ionometer (TomskAnalit, Tomsk, Russia). The ions content was determined on the device in mg/L by plotting the dependence of the emf on the ion concentration on a pre-constructed scale of standard solutions in the studied concentration range (10-1–10-4 M). A silver chloride electrode (EVL-1M3.1) was used as a reference electrode. The electrolyte content in the samples was determined by the electrical conductivity of the solution using an Expert-002 conductometer (Econix, Tomsk, Russia). The change in electrical conductivity of samples in µS g^−1^ is proportional to the concentration of electrolytes and reflects the degree of accumulation of ions in plant tissues.

### 4.13. Microscopic Analysis of Leaf Surface Structure

The third leaf from the top of each mint plant of the original and transgenic genotypes (at least three plants) was used for analysis. Fragments of the leaf midsection (2 × 3 mm) were fixed for two days in a refrigerator at +4 °C in a 2.5% glutaraldehyde solution (Merck, Darmstadt, Germany) prepared in 0.1 M Na phosphate buffer (pH 7.2) with the addition of 1.5% sucrose. Then, the leaf samples were dehydrated at +4 °C for 30 min in each alcohol solution with successively increasing concentrations: 30%, 50%, 70%, 96%, and three changes of absolute ethanol. After this, the samples were transferred to liquid CO_2_ under pressure in the device for drying at the “critical point” and slowly heated under pressure. When the pressure and temperature together passed the so-called “critical point” (31 °C and 74 bar), the pressure was reduced, thus drying the samples without any damage, bypassing the liquid–gas phase transition. Next, a thin (from 1 nm and more) metal layer was deposited onto the samples to enhance conductivity and add mechanical strength to the sample, as well as to localize the signal on the sample surface (sputtering unit SPI supplies, SPI, Santa-Clara, CA, USA). Samples were stored in closed Petri dishes with granulated silica gel. The photographs were obtained using a JSM-6380LA scanning electron microscope (JEOL Ltd., Tokyo, Japan) at various magnifications. At least 300 cells of the above tissues from three independent leaves were analyzed for each experimental treatment.

### 4.14. Statistical Analysis

The results of physiological and biochemical studies carried out in at least five replicates were processed using Duncan’s tests with the corresponding errors (*p* = 0.05). The compliance of the normality of the distribution was performed by the Kolmogorov–Smirnov method. The correlation coefficients were calculated using the Spearman method since not all analysis results had a normal distribution. Validation (significance) of the correlation coefficients was carried out by comparison with the standard values of Student’s *t*-test at *p* = 0.01 and *p* = 0.05 significance levels.

## 5. Conclusions

The use of heterologous expression of both the Fe-dependent superoxide dismutase gene, responsible for increasing hydrogen peroxide synthesis, and the bacterial choline oxidase gene, responsible for the synthesis of the osmotic glycine betaine, as well as for a non-specific increase in hydrogen peroxide synthesis, can prevent profound metabolic damage in the leaves of such a hypersensitive glycophyte as peppermint. We demonstrated that constitutive gene expression maintained growth and metabolism at NaCl concentrations such as 100 mM and 150 mM, which critically halted the development of non-transgenic mint. However, this technology is only applicable in cases where the effects of salinity can be mitigated, for example, by irrigation with fresh water. This method cannot significantly improve the productivity of mint and other plants under high-salinity conditions; it only makes the plants more resistant by mitigating damage.

## Figures and Tables

**Table 1:** *. Effect of salt stress on changes in the composition of fatty acids in mint leaves.

Lines	Fatty Acids. mol.%	Days		
		0	2	7
54WT	16:0	15.7 ± 1.11	16.7 ± 0.91	17.8 ± 2.58
	16:1n-7	2.7 ± 0.04	2.0 ± 0.16	2.1 ± 0.08
	18:1n-9	14.7 ± 2.28	13.5 ± 0.68	15.2 ± 0.41
	18:2n-6	16.0 ± 2.42	14.7 ± 0.66	18.6 ± 1.28
	18:3n-3	46.6 ± 1.00	49.1 ± 0.39	41.4 ± 3.42
	Others **	1.914 ± 0.007	1.941 ± 0.009	1.810 ± 0.083
CodA25	16:0	16.8 ± 0.93	13.5 ± 1.20	18.2 ± 1.34
	18:1n-9	13.1 ± 0.83	17.8 ± 5.45	17.9 ± 0.38
	18:2n-6	15.3 ± 0.45	13.0 ± 1.72	18.6 ± 0.54
	18:3n-3	48.4 ± 2.67	50.3 ± 2.91	37.4 ± 1.72
	Others **	1.937 ± 0.06	1.985 ± 0.062	1.724 ± 0.042
CodA8	16:0	15.2 ± 1.16	14.8 ± 0.11	18.1 ± 0.01
	18:1n-9	17.1 ± 2.67	17.2 ± 3.64	17.6 ± 0.11
	18:2n-6	15.7 ± 1.03	14.4 ± 0.69	18.4 ± 0.40
	18:3n-3	45.1 ± 0.54	47.9 ± 5.47	38.3 ± 0.80
	Others **	1.884 ± 0.01	1.935 ± 0.104	1.738 ± 0.014
FeSOD11	16:0	14.3 ± 0.33	15.9 ± 0.88	18.0 ± 0.11
	18:1n-9	20.6 ± 0.72	17.3 ± 1.73	21.4 ± 1.99
	18:2n-6	14.6 ± 0.04	15.2 ± 0.30	18.7 ± 0.78
	18:3n-3	43.2 ± 0.52	46.4 ± 2.36	33.8 ± 2.47
	Others **	1.846 ± 0.009	1.906 ± 0.058	1.653 ± 0.061
FeSOD20	16:0	14.7 ± 0.35	16.3 ± 2.00	16.0 ± 0.76
	18:1n-9	19.4 ± 0.34	18.5 ± 3.41	22.0 ± 1.05
	18:2n-6	16.9 ± 0.01	16.4 ± 0.40	16.6 ± 0.75
	18:3n-3	41.8 ± 0.62	42.8 ± 1.64	37.4 ± 2.84
	Others **	1.84 ± 0.017	1.84 ± 0.009	1.72 ± 0.058

* the table shows only the main fatty acids, the share of which exceeded 1% of the total fatty acid content, and indicates the average values and standard deviations based on the results of three independent analyses; ** includes such acids as 12:0, 14:0, 15:0, 15:1n-5, 16:1n-9, 18:0, 18:1n-7, 20:0, 20:1n-9, 21:0, 20:2n-6, 22:0, 20:3n-6, 24:0, each of which accounted for no more than 0.01–0.5% of the total; when calculating the index, minor FAs listed in the previous caption were also taken into account.

**Table 2:** Effect of salt stress on changes in the composition of volatile organic compounds in mint leaves.

Lines	VOC	Days		
		0	2	7
54 Wt	Menthone	0.68 ± 0.02	0.39 ± 0.03	0.30 ± 0.08
	Isomenthone	0.56 ± 0.01	0.36 ± 0.01	0.27 ± 0.13
	Menthol	0.93 ± 0.00	0.76 ± 0.02	0.61 ± 0.32
	Pulegone	1.07 ± 0.00	0.62 ± 0.18	1.34 ± 0.25
	Sum of all compounds *	3.38 ± 0.03	2.45 ± 0.24	2.77 ± 0,83
CodA25	Menthone	0.59 ± 0.14	0.65 ± 0.06	0.59 ± 0.38
	Isomenthone	0.55 ± 0.09	0.60 ± 0.03	0.50 ± 0.26
	Menthol	0.89 ± 0.14	0.94 ± 0.09	0.93 ± 0.37
	Pulegone	0.76 ± 0.14	0.99 ± 0.07	1.44 ± 0.37
	Sum of all compounds *	2.91 ± 0.54	3.35 ± 0,00	3.68 ± 1.39
CodA8	Menthone	0.53 ± 0.08	0.57 ± 0.03	0.64 ± 0.48
	Isomenthone	0.53 ± 0.08	0.64 ± 0.06	0.49 ± 0.25
	Menthol	1.00 ± 0.12	1.10 ± 0.08	0.92 ± 0.22
	Pulegone	0.91 ± 0.13	0.55 ± 0.03	1.36 ± 0.15
	Sum of all compounds *	3.10 ± 0.44	3.06 ± 0,17	3.67 ± 1.16
FeSOD11	Menthone	0.75 ± 0.07	0.89 ± 0.26	0.72 ± 0.36
	Isomenthone	0.55 ± 0.04	0.68 ± 0.22	0.63 ± 0.11
	Menthol	0.81 ± 0.04	0.93 ± 0.22	1.12 ± 0.05
	Pulegone	0.64 ± 0.06	1.06 ± 0.15	0.91 ± 0.28
	Sum of all compounds *	3.68 ± 1.39	2.86 ± 0.22	3.71 ± 0.74
FeSOD20	Menthone	0.96 ± 0.45	0.59 ± 0.07	0.74 ± 0.20
	Isomenthone	0.71 ± 0.27	0.51 ± 0.15	0.46 ± 0.15
	Menthol	1.03 ± 0.28	0.83 ± 0.21	0.70 ± 0.21
	Pulegone	0.60 ± 0.48	0.69 ± 0.41	1.31 ± 0.05
	Sum of all compounds *	3.50 ± 1.61	2.79 ± 0.00	3.47 ± 0.57

* includes such volatile organic compounds as 3-hexenal, hexanal (E)-2,-hexenal, β-myrcene + β-pinene, limonene, isopulegol, piperitone menthyl acetate, piperitenone, (E)-caryophyllene.

## Data Availability

The original contributions presented in this study are included in the article and Supplementary Materials. Further inquiries can be directed to the corresponding author.

## Supplementary Materials

The following supporting information can be downloaded at: https://www.mdpi.com/article/10.3390/ijms272110035/s1.
