# Supplementary material for: Antioxidant Gene Overexpression Enhances Salt Tolerance in Transgenic Peppermint (Mentha × piperita L.)
Source: Int J Mol Sci. 2026 Nov 9;27(21):10035. doi: 10.3390/ijms272110035 (PMC13763687; doi:10.3390/ijms272110035)
Supplement: Supplementary file 1 [file ijms-27-10035-s001.zip › ijms-4353438-supplementary.pdf]

Supplementary Materials for article:

Antioxidant Gene Overexpression Enhances Salt Tolerance in Transgenic Peppermint (***Mentha × piperita*** L.)

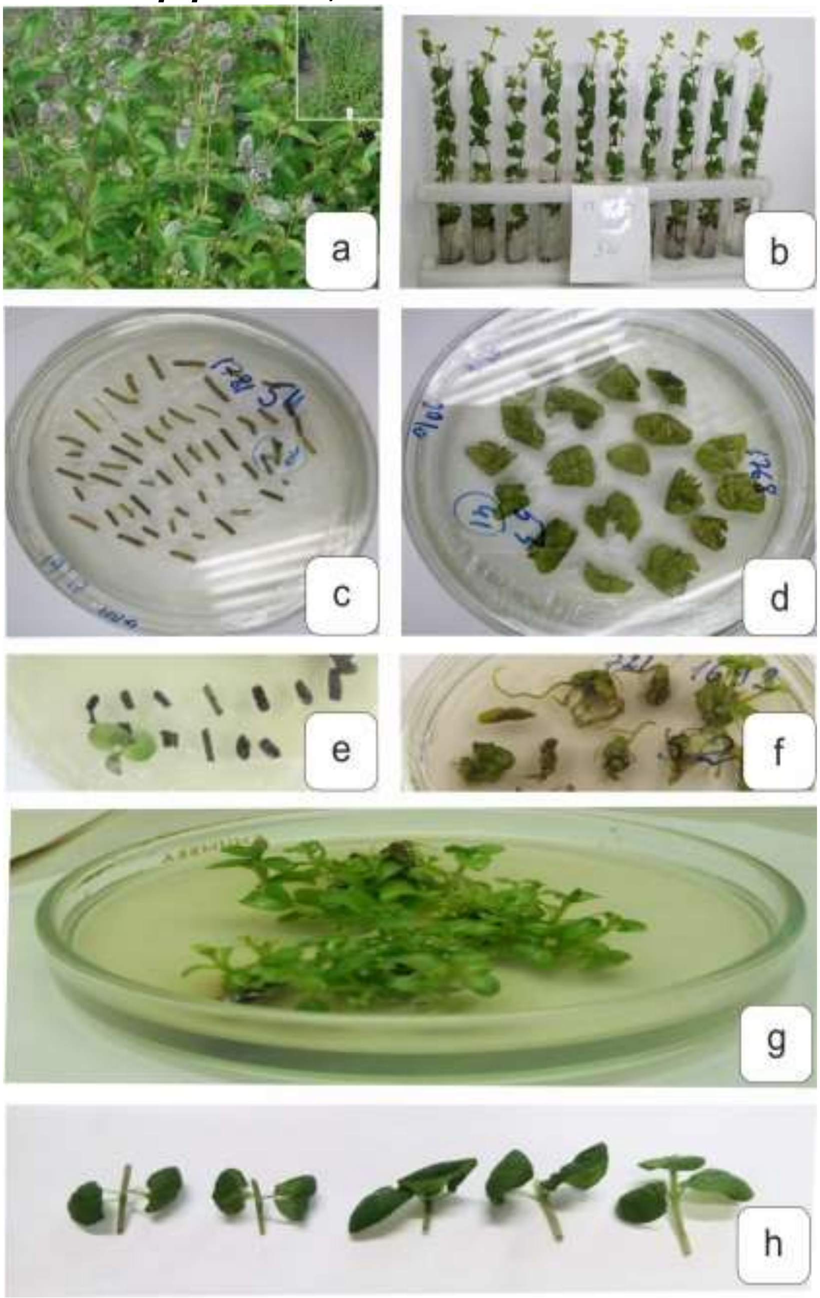

Figure S1. Obtaining aseptic culture of peppermint plants, clonal micropropagation of mint plants of the Mitchamskaya variety, preparation of explants and Agrobacterium-mediated transformation. Peppermint cv. Mitchamskaya plants under field conditions (a); clonally propagated plants after introduction into culture (b); Explants for Agrobacterium-mediated transformation from stem segments – c and leaf fragments – d; regenerants on a medium with kanamycin – e,f; cultivation of selected lines of transgenic plants before clonal micropropagation – g; explants used for propagation of transgenic lines in vitro – h.

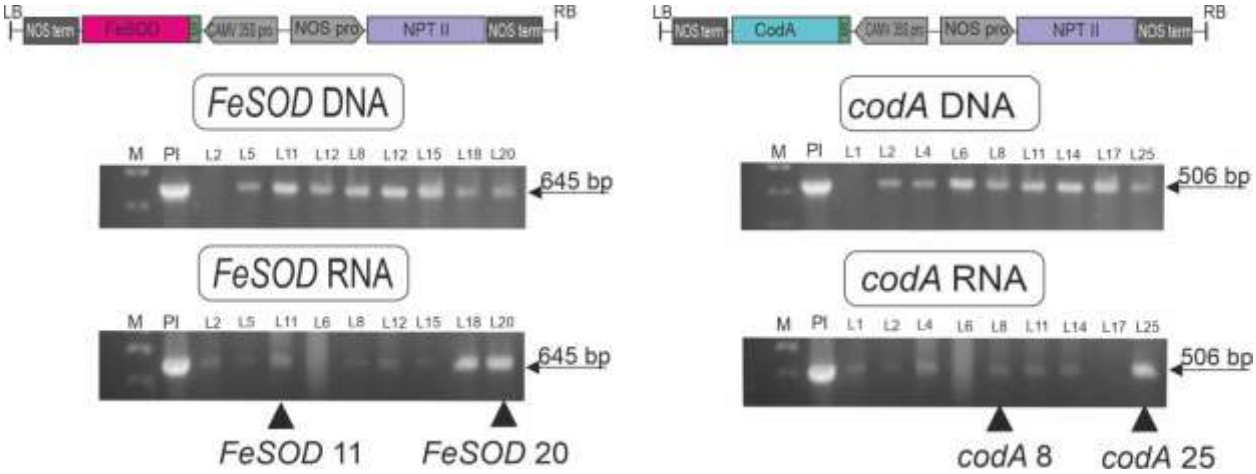

Figure S2. Assessment of the insertion presence and expression of *FeSOD* and *codA* transgenes in mint regenerants obtained by Agrobacterium-mediated transformation. The size of the amplified fragment is indicated by the arrow. M27 SibEnzyme.

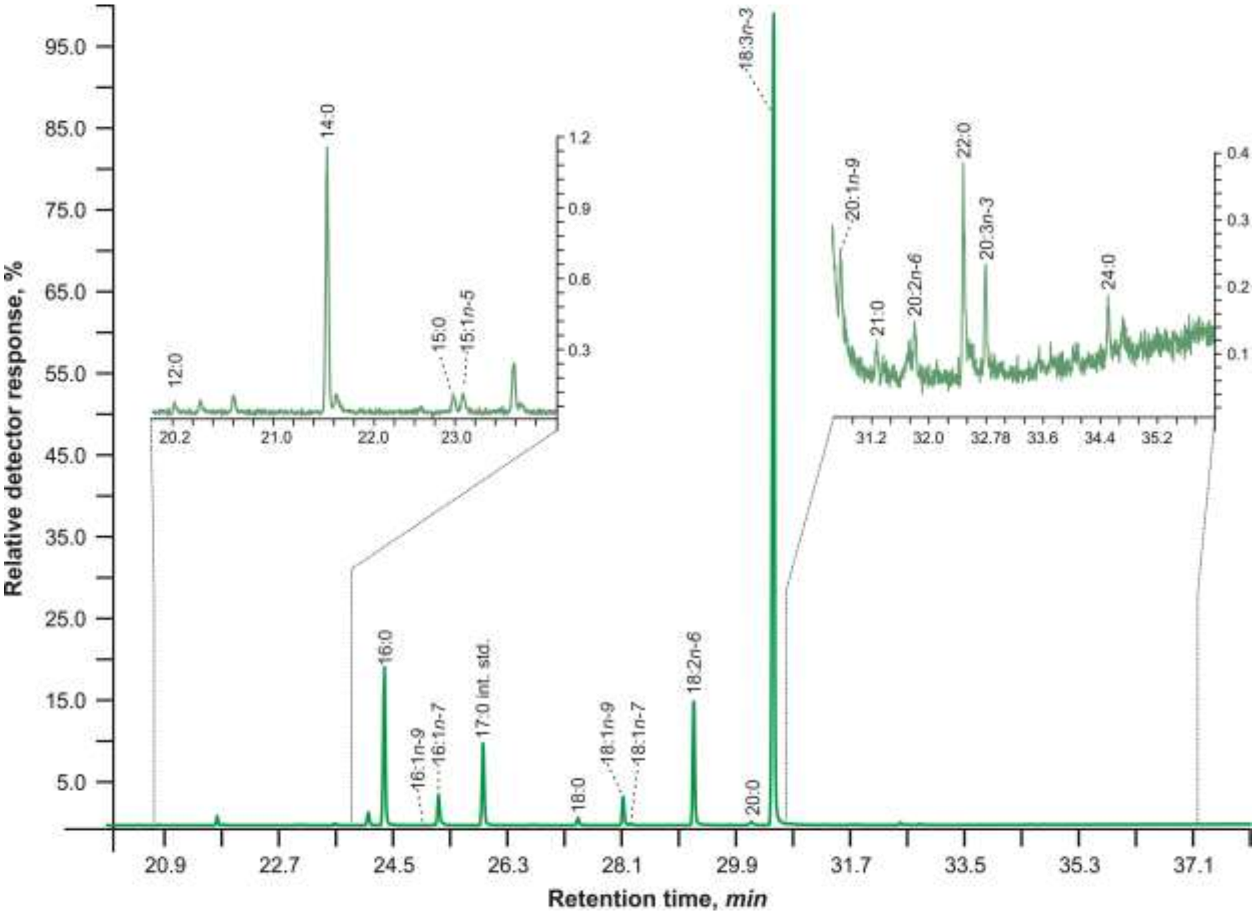

Figure S3. GC-MS chromatogram of the separation of fatty acid methyl esters obtained from total mint leaf lipids (54, wild type), total ion current. The insets show enlarged scales at 20-24 and 30.7-36.0 minute intervals, respectively.

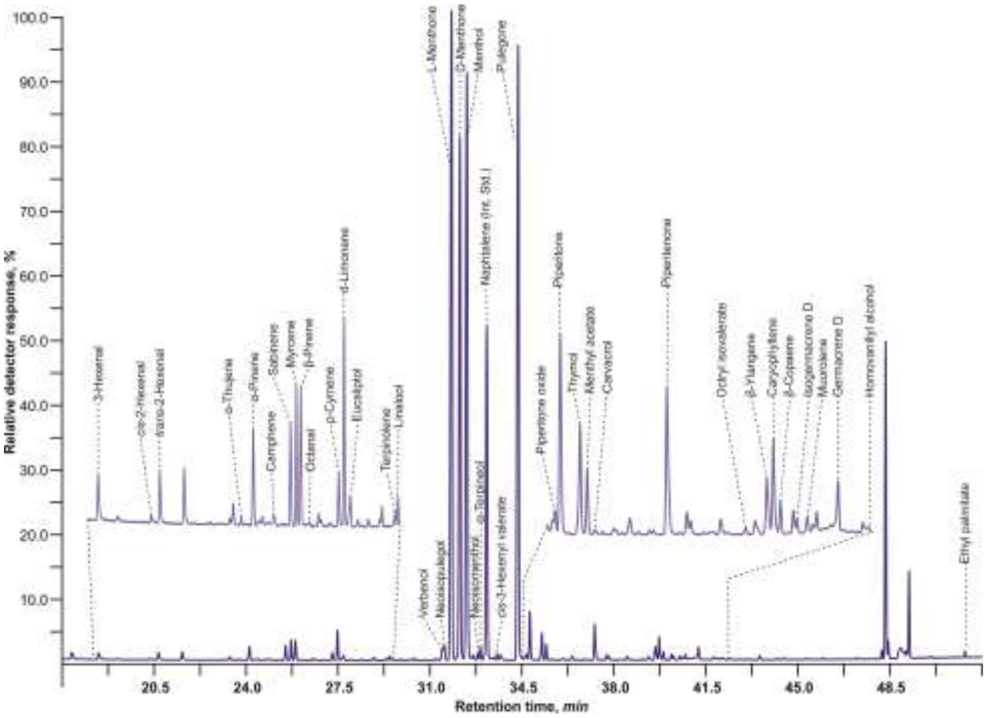

Figure S4. GC-MS chromatogram of the separation of volatile organic compounds extracted from fresh mint leaves 54 (wild type), total ion current.

Table S1. Composition of volatile organic compounds in mint leaves by experimental variants*

RI	RT	Compound Name	54			CodA			FeSOD		
995	25.86	*beta*-Pinene	0.2	±	0.06	0.3	±	0.05	0.3	±	0.26
995	25.90	3-Octanol	0.2	±	0.11	0.3	±	0.02	0.3	±	0.26
1035	27.29	*para*-Cymene	0.1	±	0.37	0.2	±	0.05	0.2	±	0.14
1161	31.45	*trans*-Verbenol	0.3	±	0.23	0.4	±	0.10	0.3	±	0.17
1163	31.54	Isopulegol	0.6	±	0.19	0.7	±	0.06	0.6	±	0.09
1171	31.79	*l*-Menthone	15.0	±	0.23	17.4	±	0.01	17.1	±	0.38
1182	32.11	*d*-Menthone	12.5	±	0.02	14.5	±	0.12	14.4	±	0.23
1190	32.37	Menthol	23.5	±	0.16	22.4	±	0.13	24.4	±	0.09
1198	32.64	Neoisopulegol	0.1	±	0.30	0.2	±	0.08	0.1	±	0.22
1204	32.82	Neoisomenthol	0.1	±	0.51	0.2	±	0.44	0.2	±	0.01
1207	32.91	*alpha*-Terpineol	0.3	±	0.20	0.3	±	0.03	0.3	±	0.13
1228	33.53	*cis*-3-Hexenyl valerate	0.1	±	0.13	0.1	±	0.02	0.1	±	0.09
1256	34.35	Pulegone	41.4	±	0.01	34.7	±	0.14	33.7	±	0.23
1271	34.80	Piperitone	0.6	±	0.19	0.6	±	0.14	0.6	±	0.21
1287	35.26	Thymol	0.6	±	0.08	0.5	±	0.08	0.6	±	0.11
1358	37.26	Piperitenone	1.6	±	0.14	1.4	±	0.01	1.3	±	0.22
1444	39.57	*beta*-Ylangene	0.4	±	0.16	0.4	±	0.03	0.4	±	0.48
1450	39.72	Caryophyllene	0.4	±	0.04	0.5	±	0.16	0.5	±	0.86
1508	41.22	Germacrene D	0.3	±	0.13	0.4	±	0.13	0.3	±	0.34
		Others**	1.7	±	0.17	4.7	±	0.35	4.4	±	0.24

*The table only lists compounds with a mass fraction greater than 0.2%

**other minor volatile compounds with a mass fraction from 0.01 to 0.2%

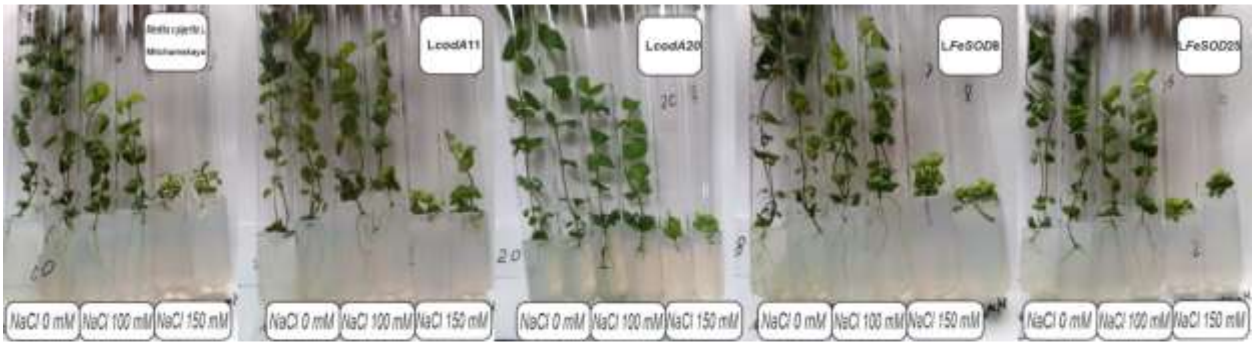

Figure S5. Growth and development characteristics of peppermint plantlets of the Mitchamskaya variety and transgenic mint lines expressing the FeSOD and CodA genes obtained by Agrobacterium-mediated transformation during in vitro cultivation on MS medium, as well as in the presence of 100 and 150 mM NaCl. Features of growth and development of peppermint plants of the Mitchamskaya variety and transgenic mint lines with expression of the FeSOD and CodA genes obtained using agrobacterium-mediated transformation during in vitro cultivation on MS medium, as well as in the presence of 100 and 150 mM NaCl.

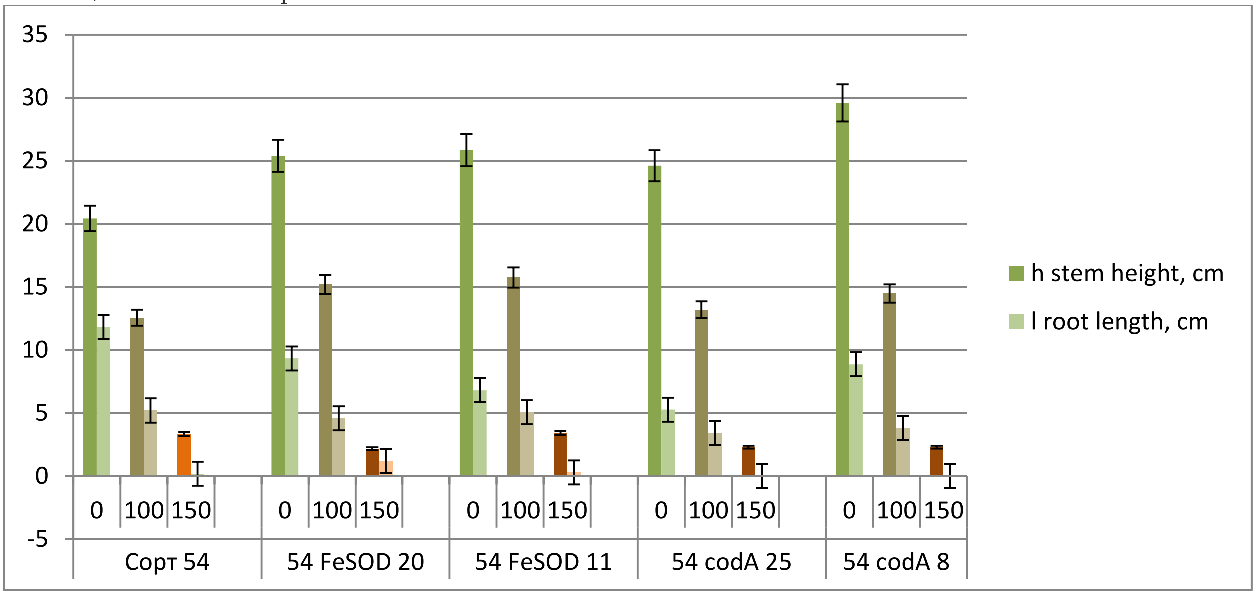

Figure S6. Characterization of the morphological parameters transgenic lines in response to 100 and 150 mM NaCl salinity in wild-type peppermint plants and *FeSOD* and *codA* transgenic lines.

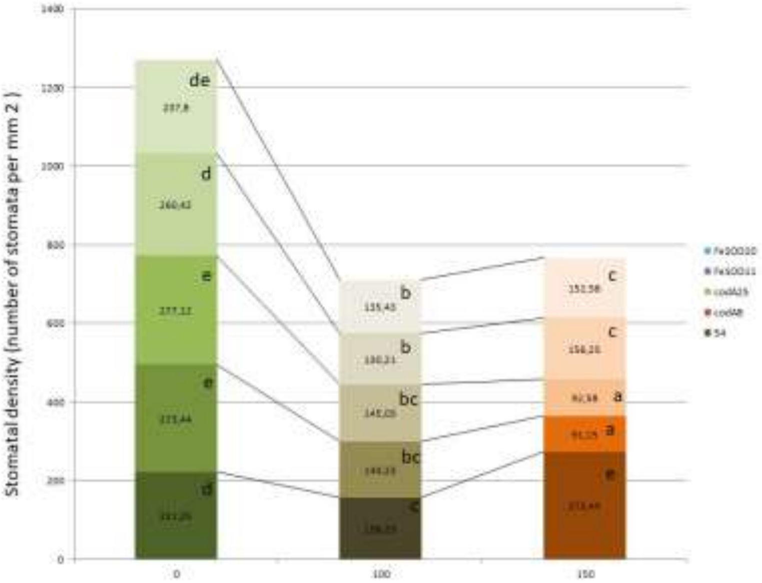

Figure S7. Density of stomata on the abaxial surface of mint leaves in wild-type peppermint line plants and *FeSOD* and *codA* transgenic lines.

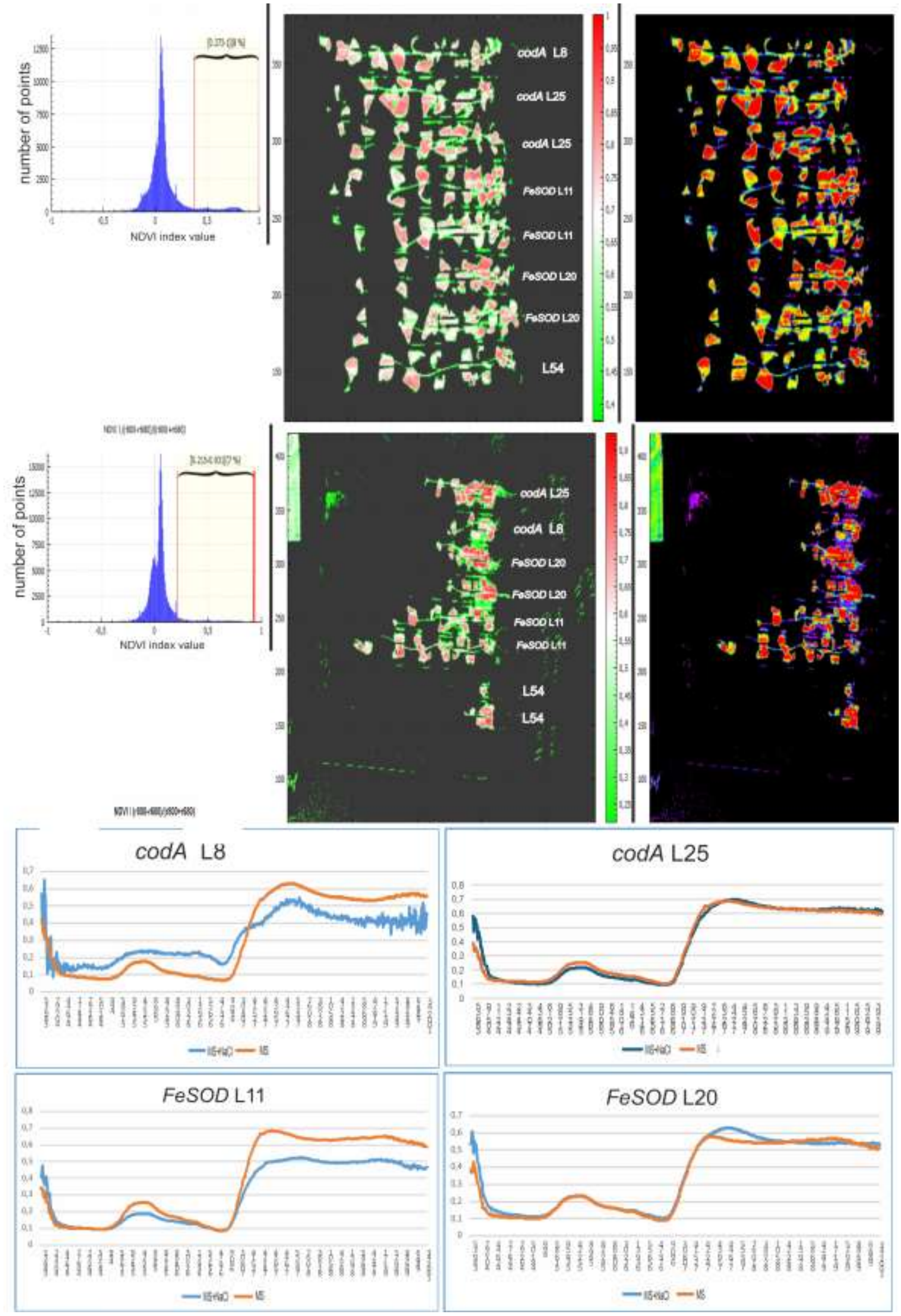

Figure S8. Visualization of the calculated NDVI index and spectral characteristics of the studied samples using hyperspectral analysis of mint leaves in wild-type peppermint line plants and *FeSOD* and *codA* transgenic lines.

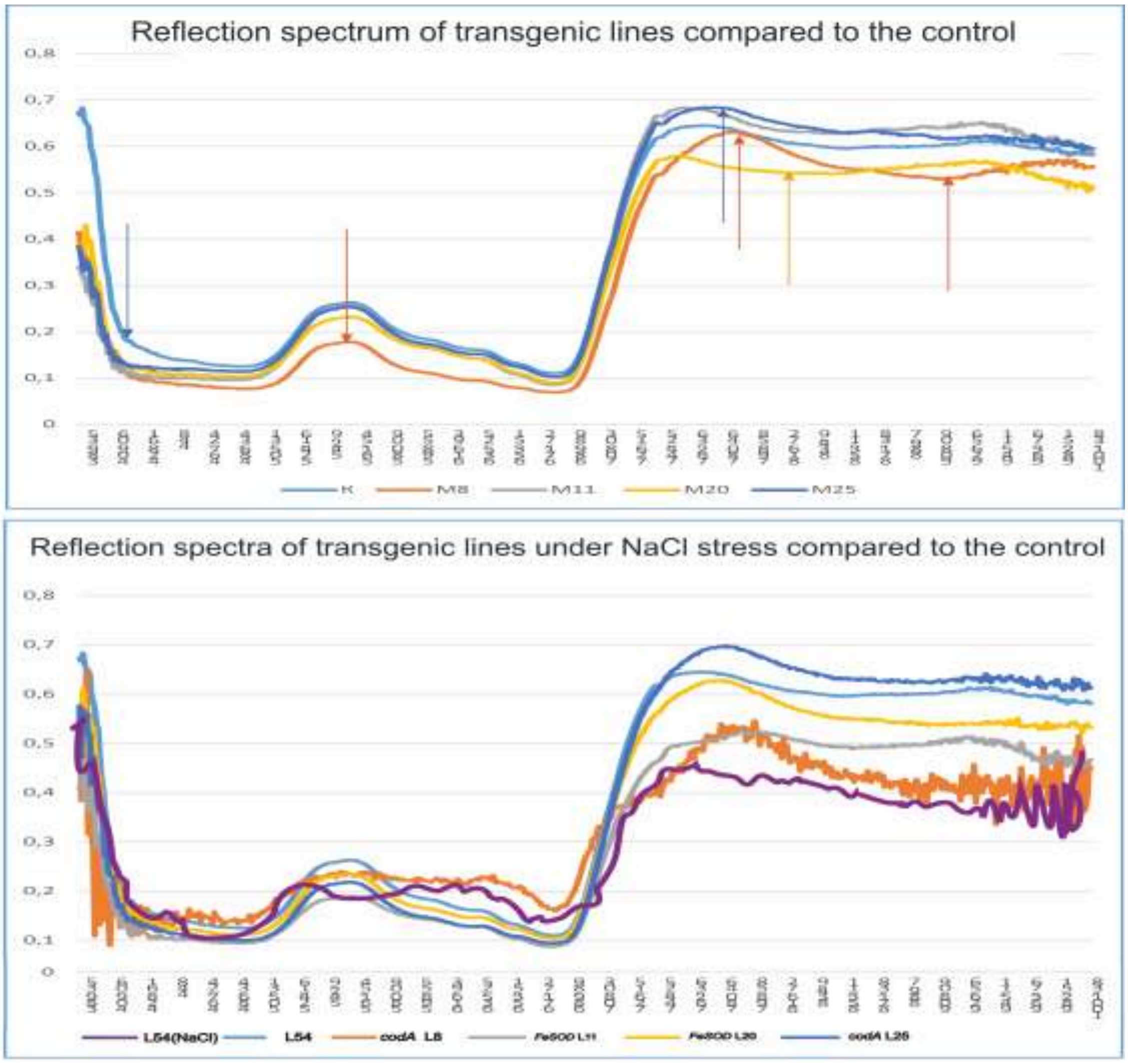

Figure S9. Spectral reflectance characteristics of the studied accessions, obtained via hyperspectral analysis of mint leaves in wild-type peppermint line plants and *FeSOD* and *codA* transgenic lines.

Comparison (ranking) of samples under saline and non-saline conditions

	1. MS									2. MS+NaCl							
	Index value					% deviation from control ?				[illegible]				% deviation from control ?			
	L54	codA L8	FeSOD L11	FeSOD L20	codA L25	M8	M11	M20	M25	[illegible]	[illegible]	[illegible]	[illegible]	[illegible]	[illegible]	[illegible]	[illegible]
NDVI	0.657	0.777	0.720	0.664	0.688	18.3	8.5	1.1	3.6	0.432	0.667	0.672	0.730	[illegible]	[illegible]	[illegible]	[illegible]
NDVI 705	0.466	0.579	0.510	0.482	0.489	24.4	9.5	3.5	1.0	0.205	0.443	0.457	0.516	[illegible]	[illegible]	[illegible]	[illegible]
g NDVI	0.418	0.522	0.456	0.433	0.450	24.9	9.1	3.6	4.1	0.290	0.450	0.433	0.497	[illegible]	[illegible]	[illegible]	[illegible]
EVI	0.937	0.863	0.960	0.866	0.949	-7.9	4.9	-5.5	7.1	0.496	0.790	0.828	0.939	[illegible]	[illegible]	[illegible]	[illegible]
EVI 800/680	0.650	0.766	0.723	0.615	0.704	17.8	11.3	-5.3	14.8	0.385	0.605	0.653	0.756	[illegible]	[illegible]	[illegible]	[illegible]
CL RED Edge	2.452	4.599	2.854	2.524	2.797	87.5	20.5	2.9	[illegible]	0.983	2.510	2.543	3.409	[illegible]	[illegible]	[illegible]	[illegible]
CI (=G)	2.332	2.524	2.933	2.506	2.411	8.3	25.8	7.5	-3.8	1.403	2.131	2.205	2.285	[illegible]	[illegible]	[illegible]	[illegible]
GM1	2.435	3.182	2.676	2.527	2.640	30.7	9.9	3.8	4.5	1.816	2.635	2.528	2.975	[illegible]	[illegible]	[illegible]	[illegible]
GM2	3.486	5.000	4.126	3.697	3.756	43.4	18.4	6.1	1.6	1.650	3.287	3.393	4.110	[illegible]	[illegible]	[illegible]	[illegible]
NWI 970/880	-0.004	0.027	-0.013	-0.017	-0.003					0.040	-0.019	-0.001	0.000				
NWI 970/850	-0.001	0.011	-0.002	-0.002	-0.008					-0.008	-0.015	-0.015	-0.005				
NPCI	-0.192	-0.150	-0.107	-0.127	-0.095	-22.0	-44.4	-33.7	-25.9	0.120	-0.147	-0.166	-0.152	[illegible]	[illegible]	[illegible]	[illegible]
PHRI	0.084	0.105	0.102	0.088	0.090					0.030	0.073	0.080	0.091				
PRI	-0.087	-0.079	-0.085	-0.072	-0.068					-0.033	-0.063	-0.064	-0.072				
PSRI	-0.025	-0.013	-0.019	-0.024	-0.019					0.059	-0.015	-0.012	-0.009				
RVI 670/680	4.561	9.960	5.885	4.920	5.065	[illegible]	[illegible]	7.9	3.6	2.136	4.764	4.476	5.805	[illegible]	[illegible]	[illegible]	[illegible]
RVI 810/680	4.692	7.644	5.926	4.898	5.261	[illegible]	[illegible]	4.4	7.8	2.456	5.026	4.851	6.173	[illegible]	[illegible]	[illegible]	[illegible]
MTCI	0.814	1.278	0.985	0.876	0.906	[illegible]	[illegible]	7.6	3.0	0.196	0.762	0.787	1.007	[illegible]	[illegible]	[illegible]	[illegible]
OSAVI 670/800	0.648	0.744	0.710	0.646	0.685	14.8	9.6	-0.3	5.9	0.435	0.642	0.652	0.718	[illegible]	[illegible]	[illegible]	[illegible]
MCARI 705/750	0.883	1.241	1.141	0.871	1.022	[illegible]	[illegible]	[illegible]	17.4	0.162	0.830	0.766	1.092	[illegible]	[illegible]	[illegible]	[illegible]
MCARI 670/700	0.117	0.077	0.150	0.112	0.122	[illegible]	28.3	[illegible]	[illegible]	0.092	0.101	0.107	0.102	[illegible]	[illegible]	[illegible]	[illegible]
TCARI 670/700	0.255	0.172	0.298	0.239	0.259	-32.7	17.1	-6.4	5.4	0.195	0.202	0.230	0.218	[illegible]	[illegible]	[illegible]	[illegible]
TCARI /OSAVI 670/800	0.394	0.231	0.420	0.368	0.378	-41.4	6.6	-6.3	[illegible]	0.449	0.315	0.352	0.305	[illegible]	[illegible]	[illegible]	[illegible]
MCARI/OSAVI 670/800	0.180	0.103	0.211	0.174	0.179	-42.6	17.2	-3.4	2.8	0.212	0.167	0.164	0.143	[illegible]	[illegible]	[illegible]	[illegible]
ARI	-0.551	-1.318	-0.950	-0.670	-0.616	[illegible]	72.5	21.7	[illegible]	0.068	-0.152	-0.365	-0.635	[illegible]	[illegible]	[illegible]	[illegible]
CRI1	3.290	6.074	4.775	4.017	3.705	84.6	45.1	22.1	-7.8	1.881	3.952	3.848	4.425	[illegible]	[illegible]	[illegible]	[illegible]
CRI2	1.716	3.072	2.773	2.128	2.134	79.1	61.7	24.0	0.3	2.186	2.803	2.328	2.888	[illegible]	[illegible]	[illegible]	[illegible]
SIPI	0.653	0.768	0.743	0.665	0.710	17.6	13.8	[illegible]	[illegible]	0.559	0.688	0.677	0.727	[illegible]	[illegible]	[illegible]	[illegible]
PSSR	3.870	6.557	4.529	3.832	4.365	69.4	17.0	-1.0	13.9	2.288	4.063	4.152	5.333	[illegible]	[illegible]	[illegible]	[illegible]
PSSR c	4.923	8.043	6.532	5.214	5.733	63.4	32.7	5.9	9.9	3.556	5.488	5.366	6.774	[illegible]	[illegible]	[illegible]	[illegible]
FRI	-1.883	-2.614	-1.216	-1.188	-0.546	[illegible]	[illegible]	[illegible]	[illegible]	-0.038	-1.898	-1.795	-2.639	[illegible]	[illegible]	[illegible]	[illegible]
ChlR 1 ?	0.700	0.780	0.660	0.673	0.672	11.4	-5.7	-3.8	-0.2	0.328	0.637	0.666	0.717	[illegible]	[illegible]	[illegible]	[illegible]
SR 705	2.743	3.753	3.081	2.861	2.918	36.8	12.3	4.3	3.0	1.515	2.562	2.660	3.133	[illegible]	[illegible]	[illegible]	[illegible]
m SR 705	0.628	0.720	0.736	0.678	0.692	14.6	17.5	8.0	2.0	0.524	0.651	0.645	0.688	[illegible]	[illegible]	[illegible]	[illegible]
SR (chl a)	0.627	0.636	0.541	0.599	0.604	1.5	-13.8	-4.5	0.8	0.650	0.595	0.624	0.618	[illegible]	[illegible]	[illegible]	[illegible]
SR (chl b)	0.142	0.092	0.119	0.116	0.136	-35.1	-16.4	-18.6	17.4	0.201	0.115	0.137	0.128	[illegible]	[illegible]	[illegible]	[illegible]
SR (chl total)	5.066	7.580	6.914	5.466	5.813	49.6	36.5	8.3	6.0	3.173	5.246	5.366	6.606	[illegible]	[illegible]	[illegible]	[illegible]
CSI 1	0.732	0.717	1.015	0.891	0.947	-2.1	38.6	21.7	6.3	1.520	0.878	0.754	0.903	[illegible]	[illegible]	[illegible]	[illegible]
CSI 2	0.214	0.141	0.167	0.200	0.193	[illegible]	-21.9	[illegible]	[illegible]	0.457	0.224	0.215	0.175	[illegible]	[illegible]	[illegible]	[illegible]
WRI	-0.008	0.032	-0.014	-0.023	0.003					0.020	-0.022	-0.003	-0.003				
RVSI	-0.069	-0.062	-0.061	-0.066	-0.075					-0.029	-0.041	-0.052	-0.060				
TVI	36.173	32.938	41.028	33.720	36.197	-8.9	17.4	-6.8	[illegible]	16.362	27.409	32.811	36.699	[illegible]	[illegible]	[illegible]	[illegible]
DSWI	2.341	2.496	2.928	2.509	2.414	6.6	25.1	7.2	-3.8	1.452	2.126	2.166	2.279	[illegible]	[illegible]	[illegible]	[illegible]
NDSI	0.173	0.267	0.183	0.180	0.194	54.1	5.5	3.7	7.8	0.073	0.188	0.164	0.220	[illegible]	[illegible]	[illegible]	[illegible]
CNLI	-0.519	-0.394	-0.515	-0.611	-0.459	-24.2	-0.9	17.6	-24.8	-0.530	-0.572	-0.490	-0.379	[illegible]	[illegible]	[illegible]	[illegible]
WBI	3.322	4.836	3.939	3.584	3.577	45.6	18.6	7.9	-0.2	1.538	3.347	3.140	4.039	[illegible]	[illegible]	[illegible]	[illegible]
Maccioni	0.647	0.758	0.639	0.629	0.666	17.1	-1.3	-2.8	[illegible]	0.517	0.640	0.666	0.708	[illegible]	[illegible]	[illegible]	[illegible]
MPRI	-0.166	-0.200	-0.213	-0.178	-0.179	20.1	28.3	6.8	0.6	-0.093	-0.146	-0.167	-0.193	[illegible]	[illegible]	[illegible]	[illegible]
RARS	4.230	6.109	5.423	4.543	4.739	44.4	28.2	7.4	4.3	2.163	4.185	4.316	5.327	[illegible]	[illegible]	[illegible]	[illegible]
PSND	0.653	0.767	0.736	0.673	0.699	17.4	12.6	3.1	3.8	0.548	0.685	0.677	0.731	[illegible]	[illegible]	[illegible]	[illegible]
MTVI	0.786	0.789	0.866	0.707	0.841	0.4	10.1	-10.1	19.0	0.449	0.634	0.748	0.846	[illegible]	[illegible]	[illegible]	[illegible]
RDVI	0.340	0.395	0.373	0.347	0.356	16.3	9.7	2.2	2.6	0.231	0.349	0.344	0.372	[illegible]	[illegible]	[illegible]	[illegible]
SPVI	0.632	0.733	0.688	0.558	0.706	16.0	8.9	-11.7	26.5	0.445	0.557	0.637	0.773	[illegible]	[illegible]	[illegible]	[illegible]
ND 750	0.068	0.118	0.056	0.057	0.070					0.067	0.099	0.067	0.104				
ND 550	0.409	0.540	0.443	0.419	0.490					0.297	0.461	0.425	0.509	[illegible]	[illegible]	[illegible]	[illegible]
NPQI	0.149	0.134	0.077	0.122	0.027	-9.9	-48.3	-18.1	-77.8	-0.079	0.167	0.165	0.139	[illegible]	[illegible]	[illegible]	[illegible]
LCI	0.423	0.561	0.485	0.436	0.458	32.5	9.0	3.0	4.8	0.177	0.437	0.416	0.498	[illegible]	[illegible]	[illegible]	[illegible]
ZTM	2.117	2.715	2.270	2.177	2.223	28.3	7.2	2.8	2.1	1.292	2.039	2.082	2.357	[illegible]	[illegible]	[illegible]	[illegible]
VOG1	1.432	1.835	1.479	1.455	1.481	14.1	3.3	1.6	1.8	1.059	1.341	1.384	1.490	[illegible]	[illegible]	[illegible]	[illegible]
VOG2	0.940	1.146	0.962	1.056	0.965	21.9	2.3	12.4	-8.6	1.439	0.858	1.050	1.086	[illegible]	[illegible]	[illegible]	[illegible]

Pairwise comparisons (salt vs. no salt, % difference)

	[illegible]	FeSOD L8-NaCl	FeSOD L8	%	codA L11-NaCl	codA L11	%	FeSOD L20-NaCl	FeSOD L20	%	FeSOD L20-NaCl	FeSOD L20	%
NDVI	[illegible]	0.432	0.777	-44.4	0.667	0.720	-7.3	0.672	0.664	1.1	0.730	0.688	[illegible]
NDVI 705	[illegible]	0.205	0.579	-64.7	0.443	0.510	-13.1	0.457	0.482	-5.3	0.516	0.489	[illegible]
g NDVI	[illegible]	0.290	0.522	-44.5	0.450	0.456	-1.3	0.433	0.433	0.0	0.497	0.450	[illegible]
EVI	[illegible]	0.496	0.863	-42.9	0.790	0.960	[illegible]	0.828	0.866	[illegible]	0.939	0.949	[illegible]
EVI 800/680	[illegible]	0.385	0.766	-49.7	0.605	0.723	[illegible]	0.653	0.615	[illegible]	0.756	0.704	[illegible]
CL RED Edge	[illegible]	0.983	4.599	-78.1	2.510	2.854	[illegible]	2.543	2.524	[illegible]	3.409	2.797	[illegible]
CI (=G)	[illegible]	1.403	2.524	-44.4	2.131	2.933	-27.3	2.205	2.506	-12.0	2.285	2.411	[illegible]
GM1	[illegible]	1.816	3.182	-42.9	2.635	2.676	[illegible]	2.528	2.527	0.0	2.975	2.640	[illegible]
GM2	[illegible]	1.650	5.000	-67.0	3.287	4.126	-20.3	3.393	3.697	-8.2	4.110	3.756	[illegible]
NWI 970/880	[illegible]	0.040	0.027		-0.019	-0.013		-0.001	-0.017	[illegible]	0.000	-0.003	
NWI 970/850	[illegible]	-0.008	0.011		-0.015	-0.002		-0.015	-0.002	[illegible]	-0.005	-0.008	
NPCI	[illegible]	0.120	-0.150	-180.0	-0.147	-0.107	[illegible]	-0.166	-0.127	[illegible]	-0.152	-0.095	[illegible]
PHRI	[illegible]	0.030	0.105		0.073	0.102		0.080	0.088		0.091	0.090	
PRI	[illegible]	-0.033	-0.079		-0.063	-0.085		-0.064	-0.072		-0.072	-0.068	
PSRI	[illegible]	0.059	-0.013		-0.015	-0.019		-0.012	-0.024		-0.009	-0.019	
RVI 670/680	[illegible]	2.136	9.960	[illegible]	4.764	5.885	[illegible]	4.476	4.920	-9.0	5.805	5.065	[illegible]
RVI 810/680	[illegible]	2.456	7.644	-67.9	5.026	5.926	[illegible]	4.851	4.898	-1.0	6.173	5.261	[illegible]
MTCI	[illegible]	0.196	1.278	-84.7	0.762	0.985	[illegible]	0.787	0.876	-10.2	1.007	0.906	[illegible]
OSAVI 670/800	[illegible]	0.435	0.744	-41.5	0.642	0.710	[illegible]	0.652	0.646	0.9	0.718	0.685	[illegible]
MCARI 705/750	[illegible]	0.162	1.241	[illegible]	0.830	1.141	[illegible]	0.766	0.871	[illegible]	1.092	1.022	[illegible]
MCARI 670/700	[illegible]	0.092	0.077	[illegible]	0.101	0.150	[illegible]	0.107	0.112	[illegible]	0.102	0.122	[illegible]
TCARI 670/700	[illegible]	0.195	0.172	13.9	0.202	0.298	[illegible]	0.230	0.239	-3.7	0.218	0.259	[illegible]
TCARI /OSAVI 670/800	[illegible]	0.449	0.231	94.8	0.315	0.420	-25.1	0.352	0.368	[illegible]	0.305	0.378	[illegible]
MCARI/OSAVI 670/800	[illegible]	0.212	0.103	105.8	0.167	0.211	[illegible]	0.164	0.174	[illegible]	0.143	0.179	[illegible]
ARI	[illegible]	0.068	-1.318	-150.5	-0.152	-0.950	[illegible]	-0.365	-0.670	[illegible]	-0.635	-0.616	[illegible]
CRI1	[illegible]	1.881	6.074	[illegible]	3.952	4.775	[illegible]	3.848	4.017	[illegible]	4.425	3.705	[illegible]
CRI2	[illegible]	2.186	3.072	[illegible]	2.803	2.773	[illegible]	2.328	2.128	[illegible]	2.888	2.134	[illegible]
SIPI	[illegible]	0.559	0.768	-27.2	0.688	0.743	[illegible]	0.677	0.665	[illegible]	0.727	0.710	[illegible]
PSSR	[illegible]	2.288	6.557	[illegible]	4.063	4.529	[illegible]	4.152	3.832	[illegible]	5.333	4.365	[illegible]
PSSR c	[illegible]	3.556	8.043	[illegible]	5.488	6.532	[illegible]	5.366	5.214	[illegible]	6.774	5.733	[illegible]
FRI	[illegible]	-0.038	-2.614	[illegible]	-1.898	-1.216	[illegible]	-1.795	-1.188	[illegible]	-2.639	-0.546	[illegible]
ChlR 1 ?	[illegible]	0.328	0.780	[illegible]	0.637	0.660	[illegible]	0.666	0.673	[illegible]	0.717	0.672	[illegible]
SR 705	[illegible]	1.515	3.753	[illegible]	2.562	3.081	[illegible]	2.660	2.861	[illegible]	3.133	2.918	[illegible]
m SR 705	[illegible]	0.524	0.720	-27.2	0.651	0.736	-11.7	0.645	0.678	[illegible]	0.688	0.692	[illegible]
SR (chl a)	[illegible]	0.650	0.636	2.1	0.595	0.541	[illegible]	0.624	0.599	4.3	0.618	0.604	[illegible]
SR (chl b)	[illegible]	0.201	0.092	118.7	0.115	0.119	[illegible]	0.137	0.116	[illegible]	0.128	0.136	[illegible]
SR (chl total)	[illegible]	3.173	7.580	[illegible]	5.246	6.914	-24.1	5.366	5.466	[illegible]	6.606	5.813	[illegible]
CSI 1	[illegible]	1.520	0.717	112.0	0.878	1.015	[illegible]	0.754	0.891	[illegible]	0.903	0.947	[illegible]
CSI 2	[illegible]	0.457	0.141	223.0	0.224	0.167	[illegible]	0.215	0.200	7.4	0.175	0.193	[illegible]
WRI	[illegible]	0.020	0.032		-0.022	-0.014		-0.003	-0.023		-0.003	0.003	[illegible]
RVSI	[illegible]	-0.029	-0.062		-0.041	-0.061		-0.052	-0.066		-0.060	-0.075	[illegible]
TVI	[illegible]	16.362	32.938	[illegible]	27.409	41.028	[illegible]	32.811	33.720	[illegible]	36.699	36.197	[illegible]
DSWI	[illegible]	1.452	2.496	-41.8	2.126	2.928	[illegible]	2.166	2.509	[illegible]	2.279	2.414	[illegible]
NDSI	[illegible]	0.073	0.267	-72.3	0.188	0.183	[illegible]	0.164	0.180	[illegible]	0.220	0.194	[illegible]
CNLI	[illegible]	-0.530	-0.394	34.5	-0.572	-0.515	[illegible]	-0.490	-0.611	[illegible]	-0.379	-0.459	[illegible]
WBI	[illegible]	1.538	4.836	[illegible]	3.347	3.939	[illegible]	3.140	3.584	[illegible]	4.039	3.577	[illegible]
Maccioni	[illegible]	0.517	0.758	[illegible]	0.640	0.639	[illegible]	0.666	0.629	[illegible]	0.708	0.666	[illegible]
MPRI	[illegible]	-0.093	-0.200	[illegible]	-0.146	-0.213	[illegible]	-0.167	-0.178	[illegible]	-0.193	-0.179	[illegible]
RARS	[illegible]	2.163	6.109	[illegible]	4.185	5.423	[illegible]	4.316	4.543	-4.9	5.327	4.739	[illegible]
PSND	[illegible]	0.548	0.767	[illegible]	0.685	0.736	[illegible]	0.677	0.673	[illegible]	0.731	0.699	[illegible]
MTVI	[illegible]	0.449	0.789	-43.1	0.634	0.866	[illegible]	0.748	0.707	[illegible]	0.846	0.841	[illegible]
RDVI	[illegible]	0.231	0.395	-41.6	0.349	0.373	[illegible]	0.344	0.347	[illegible]	0.372	0.356	[illegible]
SPVI	[illegible]	0.445	0.733	[illegible]	0.557	0.688	[illegible]	0.637	0.558	[illegible]	0.773	0.706	[illegible]
ND 750	[illegible]	0.067	0.118		0.099	0.056		0.067	0.057		0.104	0.070	[illegible]
ND 550	[illegible]	0.297	0.540	-45.0	0.461	0.443	[illegible]	0.425	0.419	1.4	0.509	0.490	[illegible]
NPQI	[illegible]	-0.079	0.134		0.167	0.077		0.165	0.122		0.139	0.027	[illegible]
LCI	[illegible]	0.177	0.561	[illegible]	0.437	0.485	[illegible]	0.416	0.436	[illegible]	0.498	0.458	[illegible]
ZTM	[illegible]	1.292	2.715	[illegible]	2.039	2.270	[illegible]	2.082	2.177	[illegible]	2.357	2.223	[illegible]
VOG1	[illegible]	1.059	1.835	[illegible]	1.341	1.479	[illegible]	1.384	1.455	[illegible]	1.490	1.481	[illegible]
VOG2	[illegible]	1.439	1.146	25.0	0.858	0.962	[illegible]	1.050	1.056	[illegible]	1.086	0.965	[illegible]

Figure S10. Detailed pairwise comparisons and spectral reflectance characteristics of the studied samples, obtained via hyperspectral analysis, including the calculation of optimal indices for *in vitro* sample assessment of mint leaves in wild-type peppermint line plants and *FeSOD* and *codA* transgenic lines.
